# A set of composite, non-redundant EEG measures of NREM sleep based on the power law scaling of the Fourier spectrum

**DOI:** 10.1038/s41598-021-81230-7

**Published:** 2021-01-21

**Authors:** Róbert Bódizs, Orsolya Szalárdy, Csenge Horváth, Péter P. Ujma, Ferenc Gombos, Péter Simor, Adrián Pótári, Marcel Zeising, Axel Steiger, Martin Dresler

**Affiliations:** 1 Institute of Behavioural Sciences, Semmelweis University, Budapest, Hungary; 2 Epilepsy Center, National Institute of Clinical Neurosciences, Budapest, Hungary; 3 Institute of Cognitive Neuroscience and Psychology, Research Centre for Natural Sciences, Budapest, Hungary; 4 Department of General Psychology, Pázmány Péter Catholic University, Budapest, Hungary; 5 MTA‐PPKE Adolescent Development Research Group, Budapest, Hungary; 6 Institute of Psychology, ELTE, Eötvös Loránd University, Budapest, Hungary; 7 UR2NF, Neuropsychology and Functional Neuroimaging Research Unit At CRCN - Center for Research in Cognition and Neurosciences and UNI - ULB Neurosciences Institute, Université Libre de Bruxelles (ULB), Brussels, Belgium; 8 Doctoral School of Psychology (Cognitive Science), Budapest University of Technology and Economics, Budapest, Hungary; 9 Max Planck Institute of Psychiatry, Research Group Sleep Endocrinology, Munich, Germany; 10 Centre of Mental Health, Klinikum Ingolstadt, Ingolstadt, Germany; 11 Donders Institute for Brain, Cognition and Behaviour, Radboud University Medical Center, Nijmegen, The Netherlands

**Keywords:** Neuroscience, Circadian rhythms and sleep, Computational neuroscience

## Abstract

Features of sleep were shown to reflect aging, typical sex differences and cognitive abilities of humans. However, these measures are characterized by redundancy and arbitrariness. Our present approach relies on the assumptions that the spontaneous human brain activity as reflected by the scalp-derived electroencephalogram (EEG) during non-rapid eye movement (NREM) sleep is characterized by arrhythmic, scale-free properties and is based on the power law scaling of the Fourier spectra with the additional consideration of the rhythmic, oscillatory waves at specific frequencies, including sleep spindles. Measures derived are the spectral intercept and slope, as well as the maximal spectral peak amplitude and frequency in the sleep spindle range, effectively reducing 191 spectral measures to 4, which were efficient in characterizing known age-effects, sex-differences and cognitive correlates of sleep EEG. Future clinical and basic studies are supposed to be significantly empowered by the efficient data reduction provided by our approach.

## Introduction

The frequency characteristics of sleep-dependent neuronal oscillations as recorded by scalp EEG are increasingly recognized as potent markers of aging^1–3^, health and disease^4^, typical and atypical development and maturation^5,6^, as well as of neurocognitive features of high practical relevance^7–9^. However, many of these studies are suffering from increased susceptibility to Type I error as a result of an inherently increased level of “researcher degrees of freedom”. That is, EEG data can be analysed in almost infinite different ways, by focusing on one or another specific electrophysiological phenomenon^9,10^. Instead of focusing on multiple frequencies or phenomena, our aim is to provide an overall characterization of the broadband NREM sleep EEG. Our data-driven approach is based on the statistical properties of the signal, in order to assess the intercept and the slope, as well as the most prominent/important spectral peak of the Fourier spectrum.

Evidence suggests the linear relationship between the logarithmic amplitude or power of EEG and the logarithm of frequency^11–13^. Such power law scaling is a general, state-independent feature of cortical EEG, suggesting that the Fourier spectrum can be reliably described by an approximation of the parameters of the following function:

1 $$ P\left( f \right) = Cf^{\alpha } $$

where *P* is power (*P* ≥ 0) as a function of frequency (0 ≤ *f* ≤ *f*_*Nyquist*_), C is the constant (or the intercept) expressing the overall, frequency-independent EEG amplitude (*C* > 0), whereas *α* is the spectral exponent indicating the decay rate (slope) of power as a function of frequency. Reported values for the spectral exponent are − 4 < *α* <  − 1, with lower values indicating lower arousal/(deeper) sleep^14,15,18^. That is, instead of providing 191 values for the power spectra of 0.5–48 Hz activity in bins of 0.25 Hz, the background slope of the spectrum of scale-free activity can be characterized just by two parameters (*C* and *α*). Most notably, if reliable, this function suggests that classical bandwise or binwise spectral analyses are not considering the frequency-determined nature of power values when applying statistical tests focusing on specific oscillatory phenomena. Similar views were expressed and successfully implemented for the analysis of various time series characterizing the power law scaling of brain activity in humans, some of them emphasizing the conceptual and methodological aspects^12–14,19–23^, while others focusing on sleep cycle effects, arousal and consciousness^15,16,24^, but none of them specifically targeting the issue of interindividual differences. In addition, available reports do not consider the constant (*C*) or the intercept as a variable of interest in describing sleep–wake EEG features.

Besides the spectral slope and the constant, there are further specific features of the EEG spectrum, known as spectral peaks^12,19^, which are upward deflections in the decreasing power law trend described by function (1) above. Peaks reflect oscillatory activities of specific frequencies^25^, which might prevent the reliable estimation of α if they are not considered^24,26^. In order to deliberately describe the power spectrum by taking into account its prominent peaks, we suggest the inclusion of a peak power function in the formula as follows:

2 $$P\left(f\right)=C{f}^{\alpha }{P}_{Peak}\left(f\right)$$

**Figure 1:**
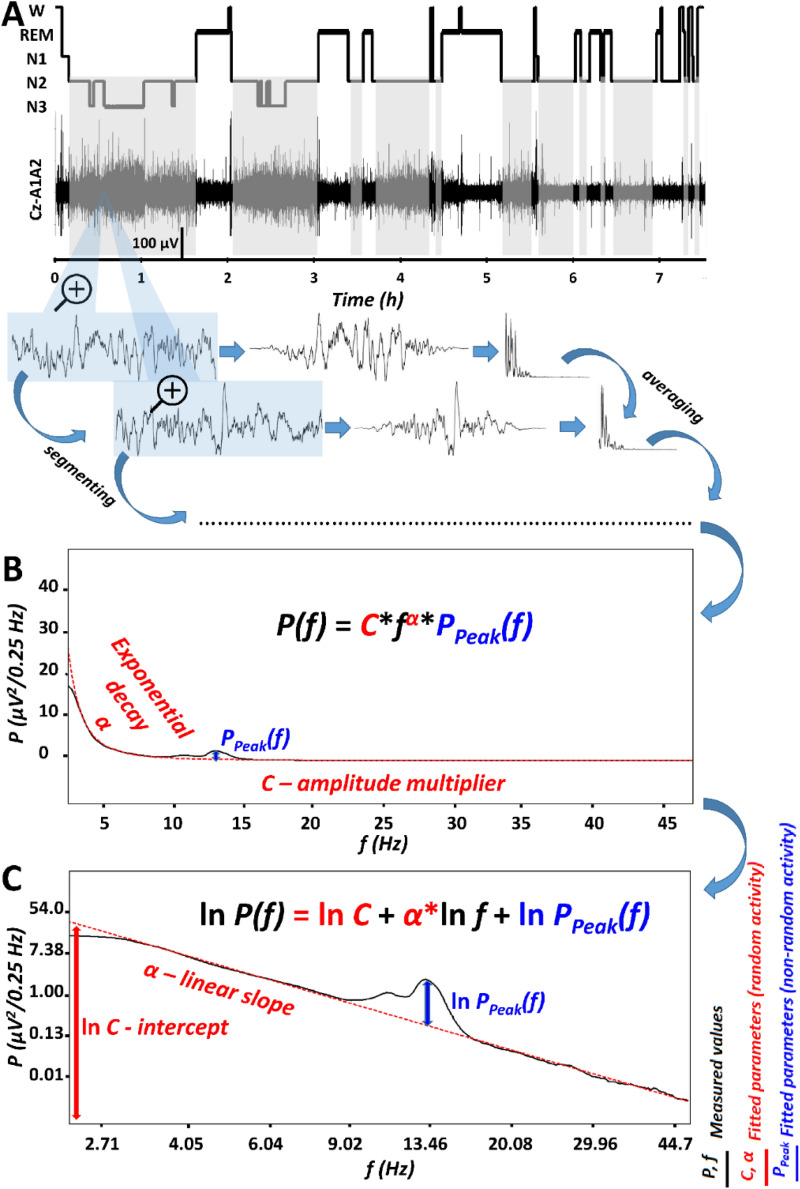
The parametrization of non-rapid eye movement (NREM) sleep electroencephalogram (EEG) spectra. (**A**) Hypnogram and steps of the spectral EEG analyses as exemplified in a representative record of a young male volunteer. Grey shaded areas represent NREM sleep, which is analysed in the present report. Blue-shaded EEG segments are magnified 4 s long epochs, with 2 s overlap and modified with a Hanning window before power spectral analysis via mixed-radix Fast Fourier Transformation (FFT). (**B**) Average spectral power (P) is characterized by a frequency (f)-dependent exponential decay (α), as well as by an overall, frequency-independent amplitude multiplier (C) and a peak power multiplier at critical frequencies [P_Peak_(f)]. (**C**) The natural logarithm of spectral power (P) is a linear function of the natural logarithm of frequency (f), characterized by a linear slope α (which is equal with α in panel **B**) and an intercept (the latter being the natural logarithm of the amplitude multiplier, C in panel **B**). In addition, this linear function has to be summed with the natural logarithm of the peak power multiplier [P_Peak_(f), equal to the same frequency-dependent function in panel **B**]. Please note that “no peak regions” can be compressed in series of all ones, resulting in reduced number of variables as compared to the bins in the original spectra.

Peak power (*P*_*Peak*_) at frequency *f* equals 1 if there is no peak and is larger than 1 if there is a spectral peak at that frequency. Thus, the number of parameters is increased by considering spectral peaks, but is still lower than the values included in the original spectra, as putative “no peak regions” can be compressed in series of all ones. It has to be noted, that *P*_*Peak*_*(f)* is a whitened power measure, because it is characterized by roughly equal power along the frequency axis and is thus statistically independent from the spectral slope (*α*) and intercept (*C*), which constitute the colored-noise or power-law noise part of the spectrum, characterized by an exponential decrease of power with increasing frequencies (Fig. 1). Although it is known that most subjects might have several peaks in their NREM sleep EEG spectra and the peak that is greatest can vary between individuals and recording locations, in the following we only consider the maximal peak in the 9–18 Hz range, emerging at a specific *f*_*maxPeak*_ frequency, with an amplitude exceeding all other potential peak amplitudes (*P*_*Peak*_*(f*_*maxPeak*_*)* > *P*_*Peak*_*(f)* for any 9 < *f* < 18). No multiple peaks are analysed in this report.

As a proof of concept, we apply these measures on a large sleep EEG dataset with previously demonstrated effects of age, sex, and general intelligence. The issue of individual differences in NREM sleep was not explicitly addressed in former reports on spectral parameters of power law scaled sleep EEG^15,16^, with the exception of a report focusing exclusively on whitened spectral peak sizes in the spindle range^27^. Our intention is to fill this gap and broaden the validity of the power law scaling-type of spectral EEG parameters, as well as to provide a set of non-redundant measures of individual differences in NREM sleep EEG.

Age was reported to correlate negatively with NREM sleep EEG slow wave activity, but positively with high frequency activity in healthy adult subjects^28^. In addition, steeper spectral slopes of wakefulness and NREM sleep-derived EEG were found in young as compared to older subjects^11,22^.

Thus, we hypothesize (H1) that the slope of the Fast Fourier Transformation (FFT)-based spectrum of NREM sleep EEG is characterized by age-dependent flattening (*α* closer to 0). In addition, aging was shown to be associated with decreased sleep spindle activity^29,30^, thus we hypothesize (H2) a negative correlation between age and spectral peak amplitude as measured by *P*_*Peak*_*(f*_*maxPeak*_*)* value. In addition to decreased spindle activity, the increase in intra-spindle oscillatory frequency (Hz) was shown to be a characteristic feature of aging according to some^29,31^, but not all^30^ reports. As a consequence, we hypothesize (H3) that maximal spectral peak amplitudes in the spindle range emerge at higher *f*_*maxPeak*_ values in aged, as compared to young subjects.

Reported sex differences in NREM and REM sleep EEG indicate higher spectral power in several frequency bands in women, as compared to men^28,32^. Such broad band and state-independent differences suggest a general tendency for a higher EEG amplitude in women, due to a contamination with non-neuronal factors, like skull thickness and bone mineral density^32,33^. As a consequence, we hypothesize (H4) that women are characterized by higher spectral intercepts, than men (*C*_*♀*_> *C*_*♂*_). Furthermore, we will reanalyze some of the reported sex differences in sleep spindle density/power, indicating increased sleep spindling in women as compared to men^28,32,34,36^, by relying on whitened spectral peak amplitudes of the spindle range (*P*_*Peak*_*(f*_*maxPeak*_*)*), the latter being a measure which is independent of overall EEG-amplitude (*C*).

Based on a largely overlapping dataset, formerly we reported another sex difference in terms of sleep spindle frequency: women were shown to be characterized by higher oscillatory frequencies as compared to men^36^. Thus, our explicit intention is to provide convergent validity of the present method, by testing the following hypothesis (H5): maximal spectral peaks occur at higher frequencies in women as compared to men (*f*_*maxPeak♀*_ > *f*_*maxPeak♂*_).

Intelligence was shown to correlate positively with NREM sleep EEG sleep spindle activity^7^. Although, a recent metaanalysis casts doubt on the sexual dimorphism of this relationship^9^, the dataset we analyse in our current report is characterized by a clear difference among women and men: women were characterized by positive correlation between sleep spindle amplitude/power and IQ, whereas null correlations were reported for men^8,36^. As our current analyses are based on the same dataset, we aim to provide convergent validity of our current method by testing the hypothesis (H6): *P*_*Peak*_*(f*_*maxPeak*_*)* values of the sleep spindle range (9–18 Hz) correlate positively with IQ in women, but not in men. Intelligence was also reported to modulate the relationship between the decrease in NREM sleep EEG slow activity associated with aging: participants showing average IQ (AIQ) scores were characterized by significant negative correlations regarding age vs. slow wave activity, whereas no such correlations were found in individuals with high IQ (HIQ) in an overlapping sample^1^. As the original report provided overwhelming evidence for an age vs relative delta power correlation as being modulated by IQ range, whereas weaker evidence was found for absolute power^1^, we do not know if this finding reflects the age-dependency of slow wave activity per se, or the combined age-dependency of slow wave activity and slow/high activity ratio. The former scenario would fit with a null effect for IQ-modulation of age vs spectral slope correlation, whereas the latter would lead to an IQ-dependence of age vs spectral slope relationship (H7).

## Results

### Goodness of fit: Is the logarithm of spectral power a linear function of the logarithm of frequency?

NREM sleep EEG spectra of 175 healthy subjects (81 females, age range 17–60 years), with a maximum of 18 available (artefact-free) common recording locations were included in our analyses. Linears were fitted to the equidistant log–log plots of the EEG power spectra below 48 Hz, excluding the 0–2 and the 6–18 Hz range, both known to be characterized by spectral peaks (slow oscillation and spindles) in NREM sleep (Fig. 1, see details in section “Methods”). The sample mean of fitted slopes (${\overline{\alpha }}$) varied between − 2.73 (SD = 0.22) and − 2.33 (SD = 0.22) for the frontocentral (Fz) and left posterior temporal (T5) region, respectively. In turn, the sample mean of the intercepts ($\overline{{\ln \;{\text{C}}}}$) varied between 3.74 (SD = 0.73) and 5.76 (SD = 0.69) for recording locations T5 and Fz, respectively (Suppl. table 1). Goodness of fit (*R*^2^) of the linear model of the equidistant 2–6 and 18–48 Hz spectral data varied in the range of 0.8955–0.9997 across subjects and EEG recording locatios. The square of the Fisher Z-transformed, averaged and back-transformed Pearson correlations between the fitted linear and the spectral data is ${\overline{\text{R}}}^{2}$ = 0.9952 (SD = 0.1578).

Here we claim that the spectral slope (*α*) and the intercept (ln *C*) carry meaningful information. In order to demonstrate that the present method of determining slope and intercept is comparable with existing methods, we tested these parameters against the respective outputs of a recently published method termed fitting oscillations & one over f (FOOOF)^23^. Our spectral slopes and intercepts correlated significantly with FOOOF slopes and intercepts, respectively. Mean correlation (Fisher-transformed, averaged and back-transformed) over recording locations was r = 0.90 for spectral slopes and r = 0.92 for intercepts (see Suppl. Figure 1).

### Spectral peaks in the 9–18 Hz range

**Figure 2:**
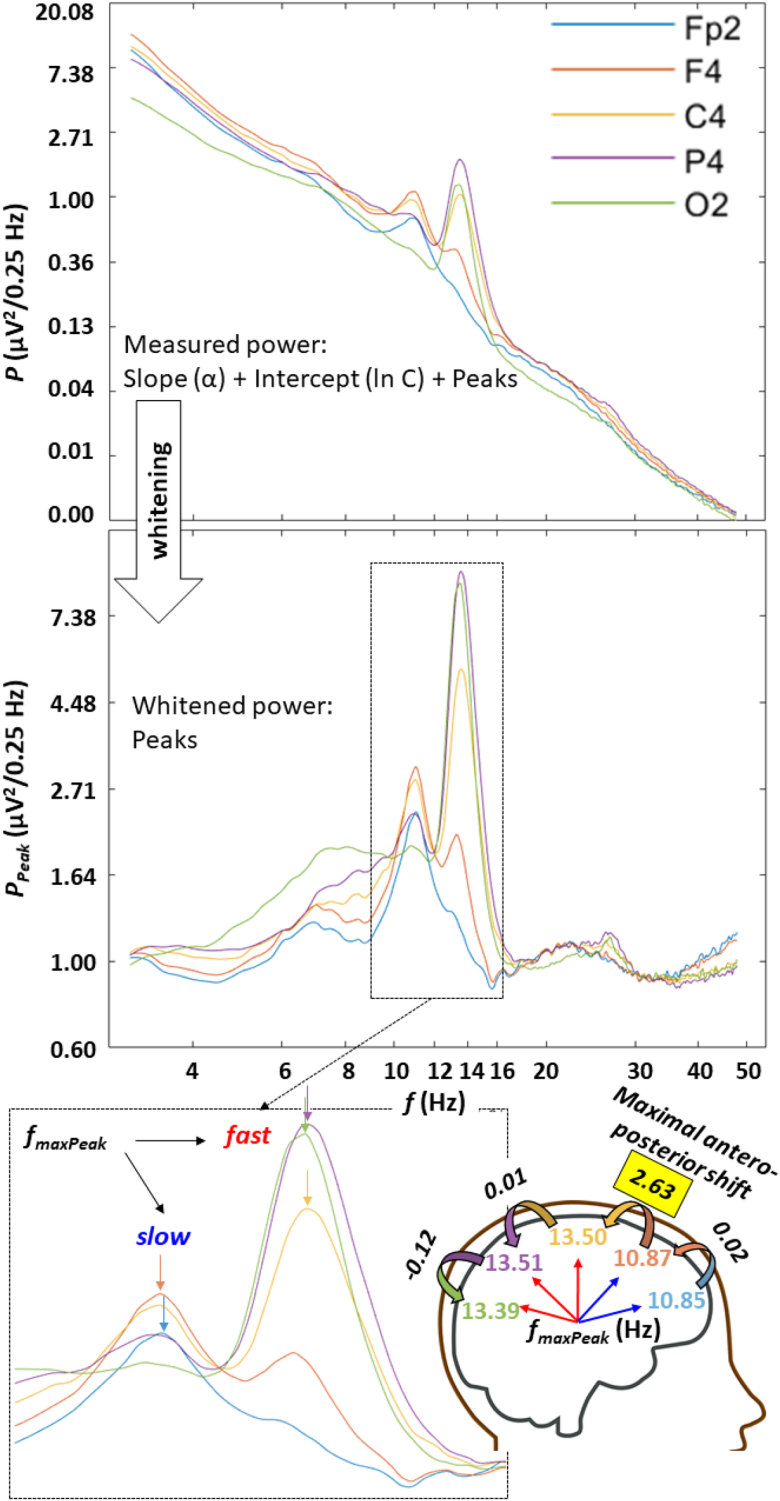
Examples for spectral peaks over the antero-posterior cortical axis in one of the subjects. Upper part: periodograms in the double natural logarithmic plane characterized by a combination of linear trends and spectral peaks. Middle panel: whitened power by subtracting the fitted linears: ln P − (ln C + α ln f); note the uniform baseline power (~ 1) and the spectral peaks. Lower panel: enlarged spectral peaks in the spindle frequency range, characterized by lower frequency maxima in the anterior as compared to the posterior recording locations (see colour-coded arrows); maximal antero-posterior shifts in peak frequency emerged between the frontal and central recording sites, demarcating slow-anterior and fast-posterior sleep spindle-related spectral peaks.

Spectral peaks in the alpha/sigma range were determined by a combination of the first and the second derivative tests indicating local maxima in mathematical terms (see details in section “Methods”). Detected peaks were ranked according to their whitened amplitude (coloured noise characterized by the spectral slope (α) and intercept (*C*) was removed before ranking). At least one peak was detected in 81.16–100% of the subjects, depending on recording location (relatively lower values were found in the temporal locations T3, and T4, whereas above 90% was the rule for other regions, see details in Suppl. Table 2). Spectral peaks with maximal amplitudes in the 9–18 Hz range were found to conform the overall topography vs frequency relationship of sleep spindles. That is, anterior spectral peaks were slower than the posterior ones (Suppl. Figure 2). The total antero-posterior frequency increase of maximal spectral peaks in the 9–18 Hz range (*f*_*maxPeak*_) equalled 1.99 Hz (sample mean). However, the above change was largely non-continuous along the antero-posterior cortical axis, as more than 83% (1.67 Hz) of the upward shift in spectral peak frequency emerged in a single, maximal value characterizing the frontal to central (54.77% of the subjects), frontopolar to frontal (36.94%), central to parietal (6.36%) or parietal to occipital (1.91%) shifts. Spectral peak frequencies (*f*_*maxPeak*_) which were detected rostral to the maximal antero-posterior upward frequency shift are hypothesized to reflect slow sleep spindles (100% of frontopolar, 63.05% of frontal, 8.28% of central, 1.91% of parietal and 0% of occipital recording sites), whereas the caudal ones are reflections of putative fast sleep spindles (0, 36.95, 91.72, 98.09, and 100% of frontopolar, frontal, central, parietal and occipital regions, respectively) (Fig. 2).

A second spectral peak with roughly half of the amplitude of the first was detected in a subgroup of subjects/EEG recording locations (6.29–50.64%) (Suppl. Table 2). However, in most of the cases the second peaks were roughly 1.5 Hz slower than the reported slow sleep spindle frequencies in an overlapping sample^36^, thus it seems that these peaks reflected rather alpha activity (~ 10 Hz) instead of true slow or fast sleep spindles (see also Suppl. Figure 2). That is the method used in the present study was robust enough in terms of the reliable detection of the dominant spectral peak of sleep spindling in the given location, but not sufficiently sensitive in testing the non-dominant sleep spindle peaks (fast spindles in the anterior and slow spindles in the posterior locations, see Suppl. Table 2 for details). A third peak was only detected in a few instances (between 1 and 6 cases, depending on recording locations, data not shown). Given the fact that we only focus on the maximal spectral peak parameters in the following parts of our paper, we can conclude that these parameters reflect the prevailing anterior slow and the posterior fast spindles, depending on recording location. However, the anatomical boundary between prevailing anterior slow and posterior fast sleep spindles varies among subjects, leading to some uncertainty in the frontal leads, which can express both slow and fast sleep spindles (in 63.05% and 36.95% of the cases, respectively).

### H1: Age-associated flattening of spectral slope

**Figure 3:**
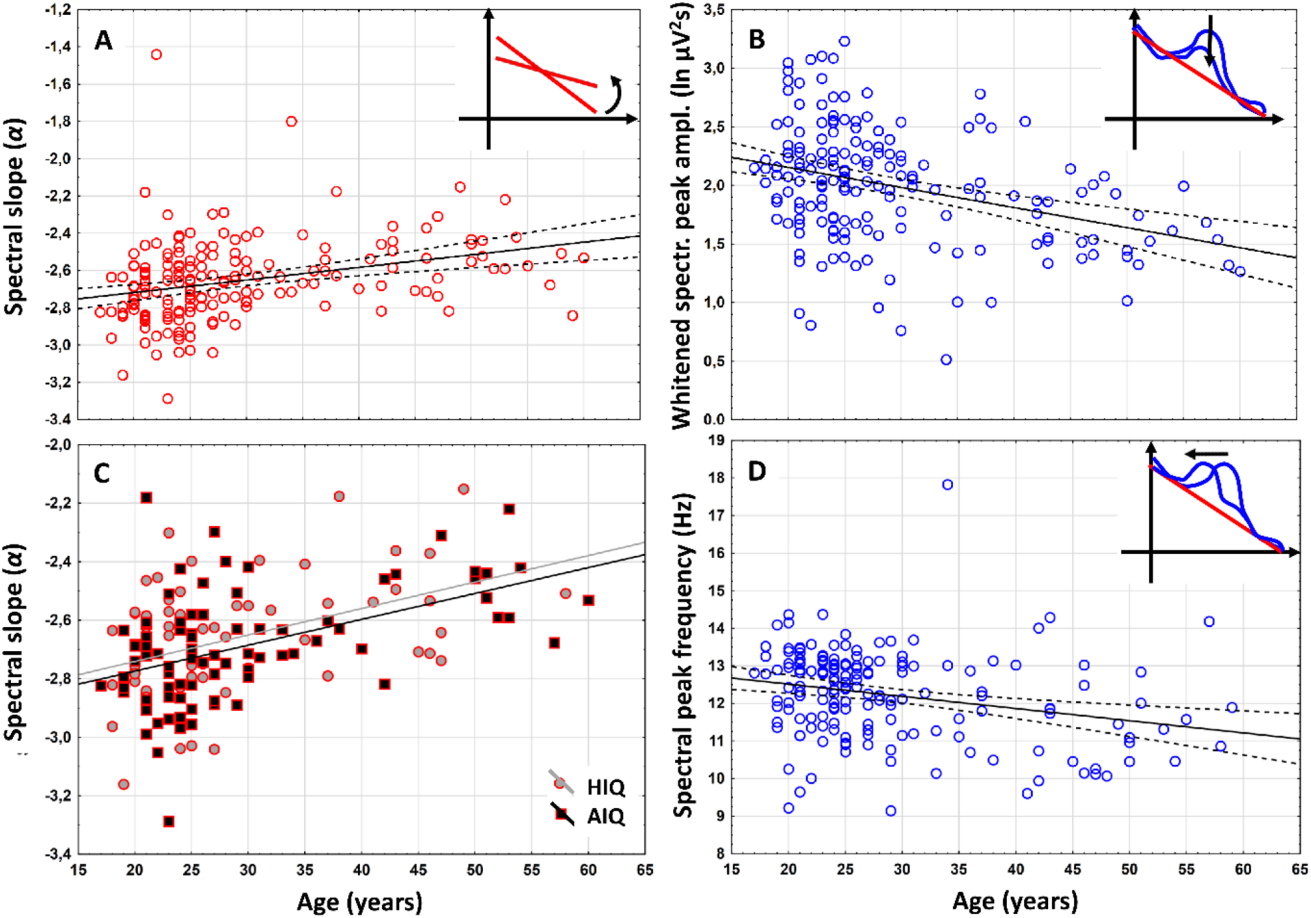
Representative scatterplots of the correlations between age and measures of the NREM sleep EEG spectra at the left prefrontal region (F3). (**A**) Correlation of age with the spectral exponent (*α*) indicating the flattening of the spectral slope in the aged subjects. (**B**) Correlations of age with the whitened maximal spectral peak amplitude in the sleep spindle frequency range (*P*_*Peak*_*(f*_*maxPeak*_). Note the decrease in whitened spectral peak amplitude in the aged. (**C**) Correlation of age with the NREM sleep EEG spectral exponent (*α*) as categorized by intelligence (*HIQ* high intelligence quotient, *AIQ* average intelligence quotient). Note the lack of an IQ effect. (**D**) Correlation of age with NREM sleep EEG maximal spectral peak frequency (*f*_*maxPeak*_) in the spindle range. Note the age-dependent decline in frequency. Color codes are consistent with Fig. 1: red—spectral slopes, blue—spectral peaks.

Positive association between age (years) and NREM sleep EEG spectral exponents (*α*), indicating age-associated flattening of slopes were found at all recording locations (Suppl. Table 3a). The Rüger’s area (consisting of spatially contingent recording locations characterized by uncorrected significances) including all recording locations in this specific case, proved to be significant at both of the new critical probability (*p*) levels (0.025 and 0.017). Thus, based on the Descriptive Data Analysis (DDA, see details in section “Methods”) procedure^37,38^, this area can be considered as a significant one (see also Fig. 3A).

### H2: Age-dependent decrease in spectral peak amplitude

Maximal whitened spectral peak amplitudes of NREM sleep EEG spindle frequencies (*P*_*Peak*_*(f*_*maxPeak*_*)*) and age correlated negatively at 10 recording locations covering the frontocentral, parietal and posterior temporal areas (F3, F4, Fz, C3, Cz, C4, T5, T6, P3, and P4). The above region remained significant after the control of multiple testing (see a representative example in Fig. 3B).

### H3: No age-related increase in spectral peak frequency was found

Maximal sleep spindle spectral peak emerge at lower *f*_*maxPeak*_ values in the frontal region of aged, as compared to young subjects. This finding evidently contrasts our prediction. Peak frequency and age correlated negatively at 8 recording locations covering the frontal and the right temporal areas (Fp1, Fp2, F3, F4, Fz, F7, F8 and T4). This Rüger’s area was significant, as all correlations conformed to both of the new critical probabilities (Fig. 3D; Suppl. Table 3c).

In order to test if changes in slow/fast spindle peak sizes could underlie these effects, that is if the maximal spindle peak “jumps” from the fast to the slow spindle peak more frequently in frontal recording sites of aged individuals, we compared the age of the following groups of subjects. Group F-Fp was characterized by a maximal antero-posterior frequency increase of *f*_*maxPeak*_ between the frontopolar and frontal recording sites, whereas for the C-F group this frequency shift was measured between the frontal and the central region. Mann–Whitney U-test revealed higher age in the C-F, as compared to the F-Fp group (U =  − 2.41; η^2^ = 0.713; *p* = 0.015). That is the age-associated dampening of *f*_*maxPeak*_ might indicate a decrease in the emergence of fast sleep spindles in the frontal region in aged subjects.

### H4: Spectral intercepts, but not peak amplitudes are higher in women as compared to men

**Figure 4:**
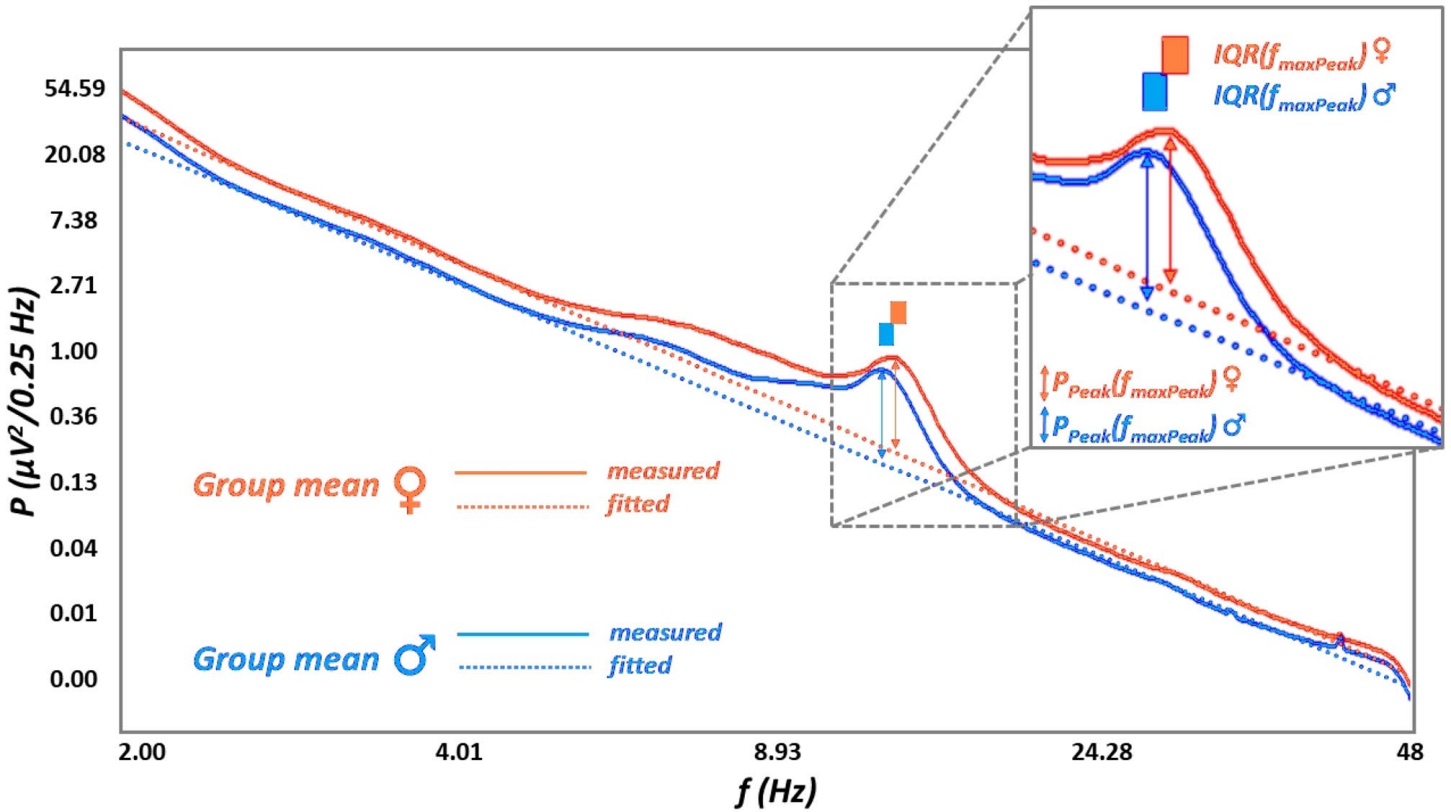
Women vs men differences in measured and parametrized mean NREM sleep EEG spectral power at electrode location C4. The natural logarithm of pectral power was averaged in women and men (continuous lines), as where individual fits (dotted lines) acoording to our current method (see details in section “Methods”). Note the overall amplitude differences (women > men), as well as the higher spectral peak frequencies (f_maxPeak_) in women and the lack of differences in spectral peak amplitudes (P_Peak_(f_maxPeak_)). *IQR* interquartile range.

The spectral intercept is the power value at which the spectral slope crosses the y-axis. Women are characterized by significantly higher spectral intercepts [the natural logarithm of *C* values in formula (1) and (2)] compared to men at all recording locations (see an example at location C4 as an example: Fig. 4).

After correction for multiple testing the Rüger-area remained significant (Suppl. table 4). As predicted women and men did not differ in NREM sleep EEG maximal spectral peak amplitudes of the spindle range (*P*_*Peak*_*(f*_*maxPeak*_*)*) at any of the recording locations (Fig. 4; Suppl. table 4b).

### H5: Women are characterized by faster sleep spindles

Women were characterized by significantly higher *f*_*maxPeak*_ values as compared to men (Fig. 4), except temporal recording locations T3 and T4. The area remained significant after the correction for multiple testing (Table 1).

**Table 1:** Women vs men differences in NREM sleep EEG spindle spectral peak frequencies.

Recording location	U (η^2^)	*p*	N_♀_	Md_♀_ (Q1–Q3)_♀_	N_♂_	Md_♂_ (Q1–Q3) _♂_	Md_♀_–Md_♂_
Fp1	1888 (.076)	***.001***	67	11.97 (11.36–12.45)	83	11.32 (10.87–11.93)	0.65
Fp2	1864 (.100)	*** < .001***	68	12.00 (11.33–12.44)	87	11.29 (10.83–11.81)	0.71
F3	2191 (.095)	*** < .001***	75	12.80 (12.10–13.25)	91	11.86 (11.14–12.86)	0.94
F4	2217 (.088)	*** < .001***	76	12.98 (12.06–13.35)	89	11.80 (11.04–12.97)	1.18
Fz	2259 (.028)	*.041*	66	13.06 (11.75–13.41)	85	12.51 (11.10–13.13)	0.55
F7	1492 (.090)	*** < .001***	59	12.23 (11.60–12.66)	78	11.59 (11.19–12.04)	0.64
F8	1608 (.088)	*** < .000***	63	12.14 (11.53–12.59)	78	11.49 (11.13–12.14)	0.65
C3	2651 (.058)	***.002***	80	13.53 (13.20–13.96)	92	13.26 (12.86–13.59)	0.27
C4	2502 (.075)	*** < .001***	79	13.60 (13.33–14.04)	93	13.28 (12.96–13.61)	0.32
Cz	1830 (.107)	*** < .001***	68	13.68 (13.39–14.13)	87	13.33 (13.05–13.64)	0.35
P3	2290 (.118)	*** < .001***	81	13.71 (13.38–14.12)	94	13.36 (13.03–13.68)	0.35
P4	2368 (.102)	*** < .001***	81	13.70 (13.37–14.12)	93	13.38 (13.06–13.68)	0.32
T3	1829 (.002)	.635	55	12.80 (12.19–13.42)	70	12.91 (11.79–13.37)	− 0.11
T4	1942 (.005)	.440	57	12.94 (12.14–13.39)	74	12.93 (11.65–13.29)	0.01
T5	1893 (.084)	*** < .001***	68	13.62 (13.27–14.06)	84	13.32 (13.00–13.63)	0.27
T6	1730 (.108)	*** < .001***	66	13.63 (13.35–14.11)	85	13.33 (12.97–13.62)	0.30
O1	2282 (.111)	*** < .001***	80	13.65 (13.34–14.10)	93	13.33 (12.96–13.64)	0.32
O2	2253 (.112)	*** < .001***	80	13.65 (13.33–14.12)	92	13.35 (12.99–13.64)	0.30

Mann–Whitney U test indicates that women as compared to men are characterized by higher *f*_*maxPeak*_ values at which spindle range *P*_*Peak*_*(f)* maxima emerge. The Rüger area containes 16 nominally significant effects. 15 of these women vs men differences were significant at both of the more stringent criteria (*p* < .025 and *p* < .017), which supports the significance of the area.

Italic and bold italic values indicate statistical significance at *p* < *.05*, and  *p* < .017, respectively.

*Md* median.

These findings might be confounded by the factor spindle type in the case if fast spindles are dominant in more anterior leads in females as compared to males. However, the analysis of the localization of the major antero-posterior frequency shift in *f*_*maxPeak*_ of women and men did not reveal a significant difference (χ^2^ = 0.42; *p* = 0.51).

### H6: IQ correlates positively with spectral peak amplitude in women

**Figure 5:**
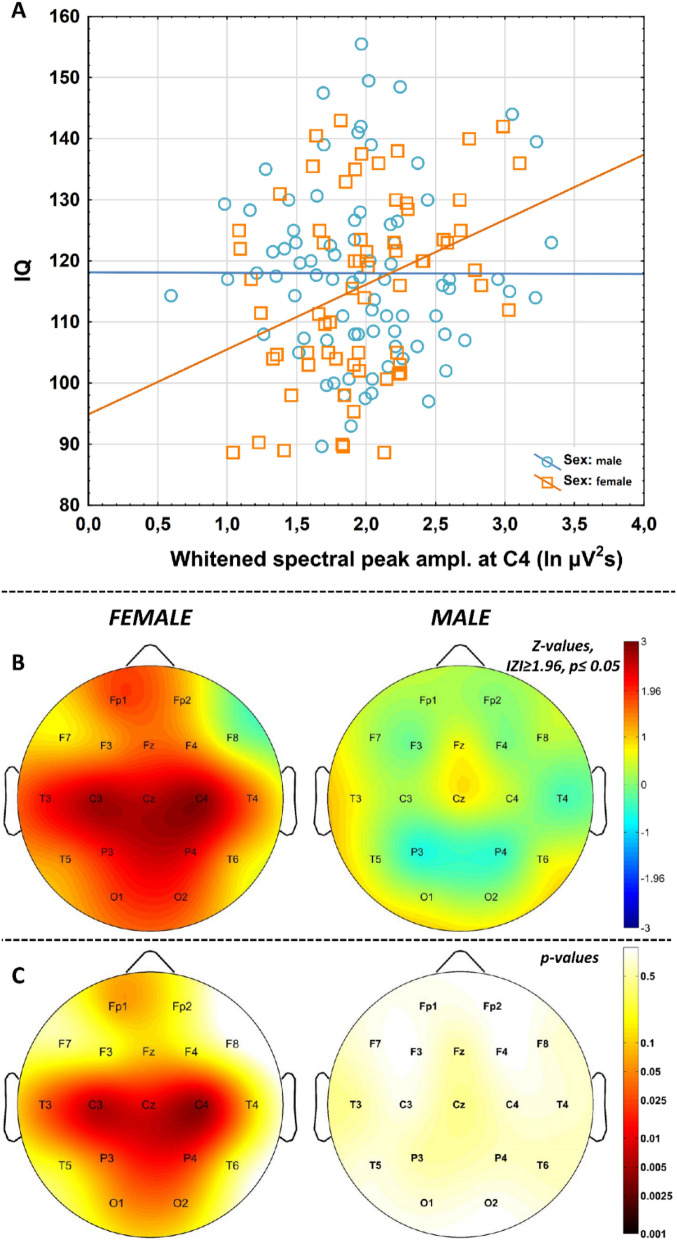
Correlations between NREM sleep EEG spindle frequency whitened spectral peak amplitudes and IQ in females and males. (**A**) Categorized scatterplot representing the correlation between whitened spectral peak amplitude of the NREM sleep EEG spindle frequency range (recording site: F4) and IQ in women and men. (**B** Pearson *r*-values were transformed to *Z*-values and represented on topographical maps. C. Significance probability maps of the correlations presented in B.

Pearson correlations revealed significant associations of whitened maximal spectral peak amplitudes (*P*_*Peak*_*(f*_*maxPeak*_*)*) pertaining to NREM sleep EEG spindle activity with IQ at recording locations C3 (N = 67, r = 0.33, *p* = 0.007), C4 (N = 66, r = 0.34, *p* = 0.005), Cz (N = 55, r = 0.34, *p* = 0.010), P3 (N = 68, r = 0.26, *p* = 0.031), P4 (N = 68, r = 0.28, *p* = 0.020), and T3 (N = 45, r = 0.32, *p* = 0.031) in women (Fig. 5; Suppl. table 5). The Rüger area at this centroparietal-left temporal region remained significant after the control for multiple testing (4/6 correlations are significant at 0.05/2 and 3/6 correlations at 0.05/3). No significant correlations of whitened spectral peak amplitude and IQ were found in men.

### H7: Do age-related flattenings of spectral slopes differ among subjects with average and high IQ?

As already presented in the former subheadings (H1) an age-associated flattening of spectral slopes characterizes the NREM sleep EEG of adult volunteers. This effect was separately assessed in subjects with average and high IQ, and results were compared. Age and slopes of the NREM sleep EEG spectra (*α*) were significantly associated in both subgroups (AIQ and HIQ). We found no significant difference between these correlations, however (Table 2). That is, age-associated flattening of the slopes of the NREM sleep EEG spectra are independent of the subjects’ IQ (Fig. 3C).

**Table 2:** Comparison of the correlations between age and the slope of the NREM sleep EEG spectra in subjects with average and high intelligence (AIQ vs HIQ).

Recording location	ρ_AIQ_	p_AIQ_	N_AIQ_	ρ_HIQ_	p_HIQ_	N_HIQ_	p_difference_
Fp1	.44	*** < .001***	79	.40	***.001***	60	.787
Fp2	.44	*** < .001***	85	.45	** < .001**	63	.901
F3	.48	*** < .001***	84	.41	***.001***	64	.622
F4	.52	*** < .001***	83	.42	***.001***	64	.476
Fz	.57	*** < .001***	70	.45	** < .001**	60	.370
F7	.39	***.001***	70	.45	** < .001**	58	.660
F8	.45	*** < .001***	69	.43	***.001***	59	.900
C3	.44	*** < .001***	84	.45	** < .001**	64	.956
C4	.45	*** < .001***	85	.43	** < .001**	64	.896
Cz	.47	*** < .001***	70	.37	***.004***	60	.507
P3	.39	*** < .001***	85	.42	***.001***	64	.801
P4	.42	*** < .001***	85	.41	***.001***	64	.927
T3	.43	*** < .001***	70	.49	** < .001**	59	.640
T4	.51	*** < .001***	70	.42	***.001***	60	.507
T5	.32	***.007***	70	.45	*** < .001***	58	.412
T6	.40	***.001***	70	.42	***.001***	60	.896
O1	.31	***.004***	85	.40	***.001***	64	.549
O2	.34	***.002***	84	.41	***.001***	64	.610

Spearman rank correlations (ρ) were significant in both intelligence groups, however, the differences between the higher (HIQ) and average (AIQ) intelligence groups was not significant (p_difference_). Bold italic values indicate statistical significance at *p* < .017.

### Overcoming model redundancy by determining the alternative intercept of the spectra

Although our model resulted in good fit with empirical data in terms of background (scale-free) activity and the majority of our hypotheses (including the ones regarding peak power features) were supported by parameters derived from Eq. (2), the spectral slope and the intercept are far from being independent in statistical terms. That is, although women vs men differences emerged in our spectral intercepts (ln *C*_*♀*_ > ln *C*_*♂*_) as predicted in H4 (see Suppl. table 4), and no sex differences in NREM sleep EEG spectral slopes (*α*) were observed (Suppl. table 6a), the intercepts and the slopes are negatively correlated in our database (Suppl. table 6b): subjects with steeper spectral slopes (lower *α* exponents) are characterized by higher intercepts (apparently higher EEG amplitudes). This might reflect the position of the intercept, which is at ln *f* = 0 (*f* = 1 Hz). The interpolated 1 Hz power (based on the fitted line in the double logarithmic plots) partially reflects the steepness of the slope of the spectrum.

**Figure 6:**
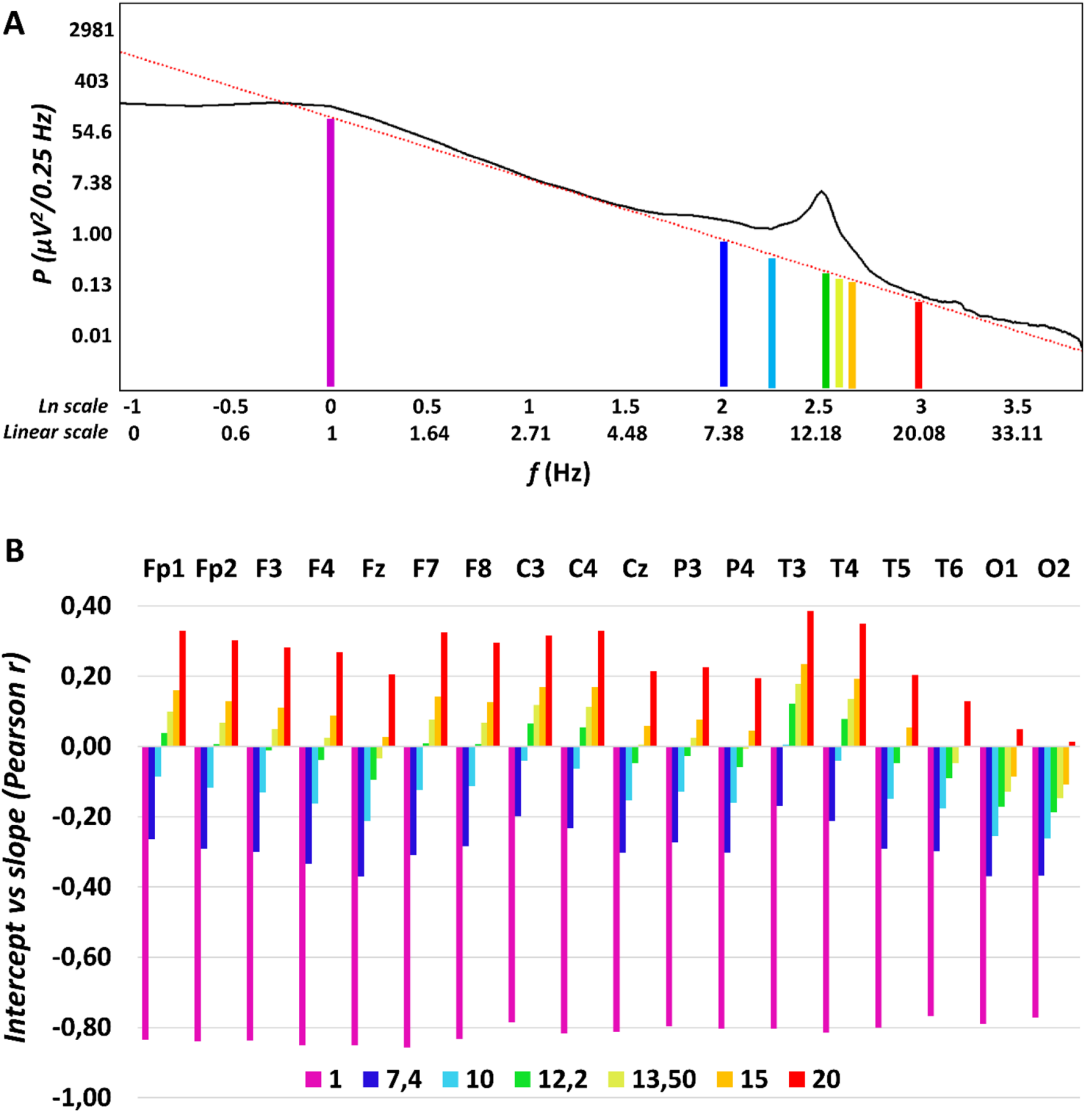
Determining the optimal alternative intercept for the NREM sleep EEG spectra. (**A**) Linear fitted to the double logarithmic plot of an average NREM sleep EEG spectral power (*P*) derived from right frontopolar location (Fp2) in a young female volunteer. Beside the original, violet-coloured intercept at ln *f* = 0 (*f* = 1 Hz), alternative intercepts are depicted at 7.4, 10, 12.2, 13.5, 15 and 20 Hz. (**B**) Between-subject correlations of the potential intercepts (ln *C*) with the slopes of the spectra (α) in a location-dependent manner. Note the negative correlations for low and the positive correlation for high frequencies, respectively. Zero-correlations are seen in the middle of the sleep spindle frequency range (at 12.2 and 13.5 Hz), although occipital recording locations are characterized by a slightly different pattern.

In order to overcome the above issue of parameter-interdependency, we derived alternative intercepts with the aim of determining parts of the interpolated scale-free spectrum at which our parameter do not reflects the steepness of the slope (*α*). We based our search for this alternative intercept on two assumptions: (1) the alternative (“slope-free”) intercept is situated at the border of low and high frequency activities, delineated by the reported sleep deprivation-induced increases and decreases of spectral power, respectively; (2) intercepts below the border mentioned in point 1. correlate negatively with the spectral slopes, whereas intercepts above this border correlate positively with slopes. Extended wakefulness of human adults is known to increase the NREM sleep EEG spectral power below the sleep spindle frequencies, that is the power of 1–9, 1–12 or 1–13 Hz according to different studies^39–43^, whereas power above 10 or 13 Hz was shown to be decreased during recovery sleep^40,42,43^. Thus, we used our fitted model parameters *α* and ln *C*, as well as the modified version of formula (3), with the last term (ln *P*_*Peak*_*(f)*) omitted (see “Methods”) to determine the interpolated scale-free natural logarithm power ln *P(f)* at frequencies of *f* = 7.4, 10, 12.2, 13.5, 15 and 20 Hz corresponding to natural logarithm values of ln *f* = 2, 2.3, 2.5, 2.6, 2.7, and 3, respectively. These alternative intercepts representing different scenarios of y-axis crosses (changing the position of the y-axis) were tested for their independence from the slopes (*α*) by Pearson correlations (Fig. 6). The pattern of correlations supported our assumptions: alternative intercepts below 12.2 Hz were found to correlate negatively with spectral slopes, whereas above 12.2 or 13.5 Hz (depending on electrode location) positive correlations were found. That is the best “slope-free” intercepts in the scale-free part of the parametrized NREM sleep EEG spectra are emerging at 12.2 Hz and the 13.5 Hz for anterior and posterior recording locations, respectively (ln *C*_2.5_ and ln *C*_2.6_). The original intercept derived at ln *f* = 0 could be termed as ln *C*_0_, according to this terminology. We reanalyzed our hypothesis based on the assumption of higher intercepts in women as compared to men, which is the only hypothesis involving term *C* of the formula (H4). Substituting ln *C*_0_ with ln *C*_2.5_ and ln *C*_2.6_ resulted in increased mean effects sizes (larger intercepts in women) from $\overline{{\eta^{2} }}$ = 0.084 to $\overline{{\eta^{2} }}$ = 0.118 (both averaged over recording locations).

## Discussion

When analyzing the Fourier spectra of EEG records performed for long periods of sleep, researchers and clinicians rely on statistics. That is, the periodograms of short modified EEG segments are averaged in order to obtain the averaged spectra^44^. As a consequence, the spectral profiles are inherently statistical in nature. In coherence with former reports focusing on sleep stages and arousal^15,16^ our current approach provides a characterization of the NREM sleep EEG Fourier spectra by taking into account their inherent electrophysiological and statistical regularities based on its power law scaling properties. Our focus is on individual differences in NREM sleep and we assume that the approach we follow results in an integrated characterization of NREM sleep EEG, which is superior in terms of construct validity and accuracy. First of all, a frequency-independent amplitude measure potentially reflecting a contamination of neuronal and non-neuronal factors, like brain activity and skull anatomy, can be reliably separated and is not mixed up in power spectral values focusing on specific oscillatory phenomena. Although the natural logarithm of term *C* derived from formula (1) and (2) (ln *C*_0_) reliably reflects the hypothesized sex differences, the model could be refined by using alternative intercepts, which were independent from the slopes (ln *C*_2.5_ and ln *C*_2.6_) (Fig. 6). Thus, we were able to determine the slope free intercepts, which—according to our best knowledge – is a first explicit and successful attempt to build a non-redundant, power law scaling-based mathematical model of sleep EEG spectra. The slope free intercept might constitute an ideal normalization value for NREM sleep EEG (spectra) in future basic and clinical studies.

In addition to the spectral intercepts, the power law functions describing the sleep EEG spectra appropriately address the issue of the ratio of EEG power at different frequencies, providing a single measure (*α*), instead of several ones scattered redundantly in all frequency bins and bands. This approach was found to be effective in deriving measures of consciousness^24^, sleep stages^15^ and arousal^16^ from sleep EEG records, as well as to index aging as a function of scale free wake EEG features^22^. Here we complete these studies with the individual differences approach of NREM sleep EEG, which was suggested to be a seminal perspective of all sleep studies^45^.

Last, but not least, spectral peak amplitudes (*P*_*Peak*_*(f)*) are whitened in our approach, that is, the scale-free part of the spectrum is effectively controlled, which might enable researchers to differentiate background and oscillatory activities at specific frequencies.

The findings derived from our approach of parametrizing the NREM sleep EEG spectra clearly supports the robustness and validity of the method presented in this paper, which was inspired by studies aiming to whiten the spectral power in the sleep spindle frequency^27,46^. As predicted (H1), age correlates positively with NREM sleep EEG spectral exponents (Suppl. Table 3a), indicating that aging is associated with flattening of the Fourier spectra (i.e. exponents closer to 0) (Fig. 3). This finding coheres with reports of bandwise power spectral analyses of NREM sleep EEG, indicating decreased low and increased high frequency activity in the NREM sleep EEG of healthy aged subjects^28^. Moreover, the steepness of the slope of the linear describing the relationship between the log-amplitude and the log-frequency of NREM sleep EEG revealed the same age-dependency^11^. Thus, our method is capable of extracting spectral slope information with sufficient precision and is a valid and simple approach to be used in future (translational) studies. The slope of the spectrum is basically a measure of the constant ratio between low and high frequency activities, which was hypothesized to reflect the ratio between inhibition and excitation, the depth of sleep and/or the level of conscious awareness^15,24,47,48^. Findings might indicate that aged subjects have lower sleep depth, but might also open new avenues beyond the exclusive focus on sleep slow waves/oscillation when studying the relationship between aging and sleep. The latter point is supported by our finding on the lack of a difference in the age-dependency of the NREM sleep EEG spectral slopes in subjects with average and high intelligence (Table 2). This finding apparently contrasts the outcomes of our previous report on the significant differences in age-dependent declines in NREM sleep EEG slow wave/oscillation of average and high IQ subjects. That is in terms of NREM sleep EEG slow waves high IQ subjects tend to age at a slower pace than average IQ subjects^1^. In spite of the fact that the database we used in the two studies are the same, the methods (band-limited spectral analysis focusing on specific frequencies vs. spectral exponent extraction) yield different results. Besides trivial methodological differences (spectral power vs exponent), our current approach of excluding the 0–2 Hz range from slope-fitting might contribute to this difference. That is, our present findings indicate that average and high IQ subjects tend to age at a same pace, at least in terms of their NREM sleep EEG spectral exponents. These contrasting results indicate that our former findings are preferentially reflecting the age- and IQ-dependency of the NREM sleep EEG slow oscillatory mechanism per se, but not the scale-free activity and/or the constant ratio of slow and high frequency activities. The latter could be a subject of aging which is at least partially independent from the well characterized age-dependent decreases in slow oscillations^49^ and is equally present in average and high IQ subjects. Recent findings and considerations suggest that the spectral slope derived from an electrophysiological signal indicates the ratio of excitation and inhibition in the underlying neural tissue^47^. Thus, according to our current findings and previously published modeling data^47^ aging is characterized by a relative increase in excitation over inhibition during the state of night time NREM sleep, and this effect seems to be relatively independent from the decreased slow oscillation reported in former studies^1,49^.

Aging was also shown to be associated with decreased sleep spindle frequency activity and decreased phasic sleep spindles in former studies^30^. These findings cohere with our current report of an age-associated decrease in whitened spectral peak amplitudes of NREM sleep EEG spindle frequency range (Suppl. Table 3b). Reports suggest that the age-dependent decrease in sleep spindles recorded over the prefrontal regions mediates the cognitive decline in later ages^50^. Moreover, it was suggested that this effect reflects the disruption of thalamocortical regulatory mechanisms involved in sleep spindle rhythmogenesis^51^. Thus, our index of whitened NREM sleep EEG spectral peak amplitude in the spindle frequency range could serve as a potential biomarker of the above mentioned^50,51^ neurocognitive aspects of aging.

The age-associated increases in the frequency of sleep spindle oscillations (also known as intraspindle frequencies) were reported in several former reports^31^, although the largest study did not reveal such changes in adulthood^30^. Our present findings reveal a non-predicted decrease in maximal frontal spectral peak amplitude in the spindle frequency range of NREM sleep EEG. The range of the spindle frequency changes clearly indicate a change from the predominant fast (~ 14 Hz) to predominant slow (~ 12 Hz) sleep spindle spectral peaks during aging. That is, our finding indicates a decrease in relative frontal emergence of fast sleep spindles during aging, rather than a deceleration of sleep spindles at a rate of 0.5 Hz/decade (Fig. 3). This post-hoc assumption is supported by our additional empirical findings indicating the increasing rates of frontal slow sleep spindle dominance in the aged. In addition, the parietal recording locations, which are almost uniformly characterized by fast sleep spindle types of spectral peaks over the whole sample, do not provide any evidence for an age-dependent acceleration of intraspindle oscillatory frequencies. That is, our findings clearly do not support this hypothesis.

Women were shown to be characterized by significantly higher NREM sleep EEG spectral intercepts as compared to men. This difference is not seen in the spectral slopes and is sharpened when using the alternative (“slope-free”) intercepts (ln *C*_2.5_ and ln *C*_2.6_ instead of ln *C*_0_). To the best of our knowledge this is the first report explicitly targeting these issues. We based our hypothesis on findings suggesting that women vs men differences in EEG power are largely frequency-independent^28^, thus indicating an overall amplitude effect captured by the term *C* in formula (1) and (2). That is, previous reports focusing on specific frequency ranges and oscillatory phenomena are confounded by overall amplitude differences in the EEG of women and men. Examples for such potentially confounded findings are reports on women vs men differences in sleep spindle densities/occurrences. Spindles detected by fixed thresholds^34,35^ or raw (non-whitened) spectral power values of the spindle frequency range^28,32^ indicate sex differences (increased sleep spindle density/activity in women), but are not controlled for overall amplitude differences. It has to be noted however, that one of the early publications cited above hypothesized that women vs men differences in sleep EEG spectral power might reflect sex differences in skull thickness^32^, but—at least to our best knowledge—this hypothesis remained largely unexplored from the electrophysiological point of view. Our current approach considers this issue and provides a reliable and potentially useful method for controlling non-specific effects, potentially contaminated with non-neuronal issues in EEG amplitude. The estimation of the spectral intercept provides a simple index which can be included in future biophysical, electrophysiological-modeling studies of the skull-thickness-EEG power issue. Our current findings clearly indicate the lack of sex differences in sleep spindle power when overall amplitude women vs men differences are controlled (Fig. 4; Suppl. table 4).

Women were shown to be characterized by higher frequency sleep spindle oscillations as compared to men according to our former study based on the individual adjustment of sleep spindle frequencies and amplitudes^36^. This finding was strengthened by our current report based on the detection of whitened spectral peak location with 0.0052 Hz resolution (Table 1). That is, our current finding strengthens the validity of our spectral parametrization approach. In addition, the hypotheses suggesting that sleep spindle frequency is accelerated by either progesterone and its neuroactive, indirect GABA-agonist metabolite allopregnanolone^52^ or the progesterone-induced hyperthermia^53^ during the follicular phase of the menstrual cycle in women are indirectly supported by our present findings. Although our participants were not controlled for menstrual cycle phases and oral contraceptive use, we can assume that at least some of the female subjects were examined during the follicular phase of their menstrual cycle. Furthermore, oral contraceptive use involve the intake of progestagenic compounds, which might induce some of the neural effects of endogenous progesterone in naturally cycling women.

Here we reveal a positive correlation between whitened spectral peak amplitude of sleep spindle frequency activity during NREM sleep and IQ in women, but not in men (Fig. 5; Suppl. table 5). Intelligence was shown to be reflected in the intensity (amplitude and/or density) of phasic sleep spindle events or alternatively in the spectral power of sleep spindle frequency activity during NREM sleep^7–10,36^. In the database we use in our present study a marked sexual dimorphism of this effect was also revealed: women but not men were shown to be characterized by the sleep spindle amplitude/power vs IQ correlations^8,36^. Although this latter effect was not unequivocally reflected in a significant meta-regression between effect size and % female in the sample in a subsequent metaanalysis^9^, here we refer to it because convergent findings obtained by different methods used on the same dataset are an issue of validity of the methods. That is, we reproduced the positive sleep spindle vs. IQ correlation in women by using a linear fitting approach to the log–log spectra of NREM sleep and a concurrent whitening of spectral peaks, without assumptions on time-domain sleep spindle features. Again, this finding might strengthen our views on the reliability of the method of analyzing the constant, the slope and the (whitened) peak attributes of the NREM sleep EEG in human subjects.

Among the shortcomings of our work we would emphasize the lack of slow vs fast sleep spindle differentiation by the current version of our method, as well as the fact that we disregarded low frequency power (< 2 Hz) when fitting the slopes. Fitting of two slightly overlapping spectral peaks instead of just one, would increase considerably the complexity of the approach, whereas our intention was to keep the process as simple and intuitive as possible. Moreover, we intended to follow the already published method of finding the maximal peak in the spindle frequency range and correlating its amplitude/power with neurological-clinical and cognitive data^27,46^. Similarly, the potential and largely unpredictable contamination of low frequency power with sweating artefacts, as well as the high-pass filtering effects of gold-coated electrodes^54^ we used in our studies precluded us from a precise measurement of the power law scaling at low frequencies below 2 Hz. Our approach of excluding the alpha and spindle frequencies before fitting a linear to the equidistant double logarithmic NREM sleep EEG power spectra requires a priori knowledge on the position of the spectral peaks and, as a consequence, increases the researchers degrees of freedom. In addition, this approach inherently omits a wide range of frequencies when fitting the linear. Although, there are reported methods for handling the above issues^14^, here we focused on the current method because of our the explicit intention of comparability with former reports focusing on NREM sleep EEG power spectra and neurocognition^27,46^.

In sum, the parametrization of NREM sleep EEG of healthy adult subjects by relying on the power law scaling behavior of the electrical activity of the brain, as well as by completing this statistical property with the prominent spectral peak at the sleep spindle range, provides an integral method of describing and characterizing individual differences in sleep and cognition. Here we show, that most of the features of NREM sleep EEG can be efficiently compressed in the spectral intercepts, slopes and peaks, at least in terms of demographic (age, sex) and cognitive (IQ) correlates of sleep. It remains to be determined, if known arousal and sleep state-dependent changes^15,16^ or overnight sleep dynamics^48^ can be efficiently completed with measures of sleep regulatory mechanisms (e.g. homeostatic and circadian factors) derived from our integrative parameters of NREM sleep EEG spectra. In addition, further studies are needed for an adequate handling of multiple spectral peaks and low frequency (< 2 Hz) oscillations in the non-full-band EEG.

## Methods

### Subjects/databases

Data was combined from multiple databases (Max Planck Institute of Psychiatry, Munich, Germany; Institute of Behavioural Sciences of Semmelweis University, Budapest, Hungary) for this retrospective multicenter study^3,8^. Polysomnography data were recorded from 175 participants 81 females, 94 males, mean age 29.57 years, age range 17–60 years) and IQ scores were measured for 149 participants (68 females, 81 males, mean age 29.23 years, age range 17–60 years). Volunteers were recruited also via Mensa Germany and Mensa Hungary to increase the number of highly intelligent individuals. As some of the participants have missing data of some electrodes and/or IQ scores the data numbers from which the statistical analysis was conducted are always reported in the results.

Based on self-reports, none of the participants had a history of psychiatric or neurological disorders. Alcohol consumption was restricted before recording, but a small amount of caffeine (max. 2 cups of coffee before noon) was allowed to the participants. Based on self reports 8 participants were light or moderate smokers. Data were combined from multiple databases (Max Planck Institute of Psychiatry, Munich, Germany; Institute of Behavioural Sciences of Semmelweis University, Budapest, Hungary). The experiment was conducted in full accordance with the World Medical Association Helsinki Declaration and all applicable national laws and it was approved by the institutional review board, the Ethical Committee of the Semmelweis University, Budapest, or the Ludwig Maximilian University, Munich. Written informed consent was obtained from adults participants and parents/guardians of the children (age: 17 years).

### Psychometric intelligence

Standardized nonverbal intelligence tests were recorded from 149 participants: 70 of them completed the Culture Fair Test (CFT)^55,56^ and 39 of them completed the Raven Advanced Progressive Matrices (Raven APM)^57^ test. 40 participants completed both tests. These tests have been shown to similarly measure abstract pattern completion and are particularly good measures of the general factor of intelligence^58–60^. A composite raw intelligence test score was calculated, expressed as a Raven equivalent score (RES)^1^. RES for Raven APM tests was equal to the actual raw test score, whereas RES of the CFT test raw scores were equal to the Raven APM score corresponding to the IQ percentile derived from CFT performance and the age of the participant. Scores were averaged for participants who completed both tests. Standardization of APM was applied according to 1993 Des Moines (Iowa). Based on their mean IQ score, the sample was split into an average (AIQ: 88 < IQ < 120; $\overline{{{\text{IQ}}}}$ = 106.9; N = 85) and a high intelligence (HIQ: 120 ≤ IQ < 156; $\overline{{{\text{IQ}}}}$ = 130.38; N = 64) subgroup^1^.

### Polysomnography recordings

Detailed data recording procedures and power spectral analysis are also reported in published studies^2,8^. Sleep data were recorded on two consecutive nights by standard polysomnography including EEG, electro-occulography (EOG), electrocardiography (ECG) and bipolar submental electromyography (EMG). EEG channels were placed according to the international 10–20 system (Fp1, Fp2, F3, F4, Fz, F7, F8, C3, C4, Cz, P3, P4, T3, T4, T5, T6, O1, O2 and left and right mastoids)^61^. Impedances for the EEG electrodes were kept below 8 kΩ. The sampling frequency was either 249 Hz, 250 Hz or 1024 Hz, depending on recording site (Suppl. table 7). Data were offline re-referenced to the average of the mastoid signals and notch filtered at 50 Hz. Electrodes excluded from the analysis due to artifacts and/or recording failures were treated as missing data. The number missing data for the total 175 participants is reported in Supplementary Table 8, separately for each electrode. Recordings of the first night were used for habituation and therefore were not included in further analyses. Sleep data of the second night in the laboratory were scored for sleep-waking states and stages according to standard AASM criteria on a 20-s basis^62^ by an expert. Furthermore, artefactual segments were marked on a 4-s basis and excluded from further analyses.

### Power spectral analysis

Power spectral densities were calculated for the NREM (N2 and N3) sleep, in 0.25 Hz bins from 0 Hz to the Nyquist frequency (*f*_*Nyquist*_) by relying on 4 s Hanning-tapered, non-artefactual windows. A 50% overlap was used for consecutive windows, whereas mixed-radix FFT for calculating power spectral densities. Power spectral densities from all 4 s windows were then averaged. As data were recorded with different EEG devices producing different analog filter characteristics, average power spectral densities were corrected as follows^1^: An analog waveform generator was connected to the C3 and C4 electrode positions of all EEG devices and sinusoid signals of various frequencies (0.05 Hz, every 0.1 Hz between 0.1–2 Hz, every 1 Hz between 2–20 Hz, every 10 Hz between 10–100 Hz) were generated with 40 and 355 μV amplitudes. The amplitude reduction rate of each recording system at each frequency was determined by calculating the proportion between digital (measured) and analog (generated) amplitudes of sinusoid signals at the corresponding frequency. The amplitude reduction rate was averaged for the 40 and 355 μV at each frequency. The reduction rate at the intermediate frequencies were interpolated by spline interpolation. The measured power spectral density values were corrected with the device-specific amplitude reduction rate by dividing the original value with the squared amplitude reduction rate at the corresponding frequency according to previous suggestions^63,64^.

### Estimation of the spectral intercepts and slopes

Basically our approach is based on obtaining the power spectrum of the EEG signal (see above), fitting a line to the log–log power and performing a peak detection. In order to manage the second step the power law function (formula (2)) was transformed to one which fits in the double logarithmic plots as follows (Fig. 1C):

3 $$\mathrm{ln}P\left(f\right)= \mathrm{ln}C+ \alpha \mathrm{ln}f+ \mathrm{ln}{P}_{Peak}\left(f\right)$$

This means that the natural logarithm of spectral power (*P*) is expressed as a linear function of the natural logarithm of frequency (*f*). In addition, there are two terms in the equation: the natural logarithm of the constant (*C*) and the natural logarithm of peak power (*P*_*Peak*_, see Fig. 1). If the latter equals 1 (*P*_*Peak*_ = 1), that is, there is no peak at a given frequency *f*, the value is 0 (ln 1 = 0). The logarithmic frequency scale inherently induces increasing data density at higher frequencies. Thus, a linear fit to this data would induce a strong bias against low frequency bins, which would contribute less to the determination of slopes compared to the higher frequency bins. In order to manage this problem and obtain an equal distribution of the data points, power values were interpolated up to the smallest frequency step (0.0052 Hz) by the piecewise cubic Hermite interpolation method. In the next step a linear was fitted to the 2–48 Hz frequency range of this equidistant log–log plot, excluding the 6.0052–17.9948 Hz frequency range corresponding to the alpha and spindle bands (in order to avoid those parts of the NREM sleep EEG spectra which are characterized by oscillatory activities as well). This part of our procedure was inspired by two former studies using a similar approach for whitening of the NREM sleep spindle spectra^27,46^. The slope of the linear is *α*, whereas its intercept is ln *C*.

Our intention to determine alternative, slope-free intercepts of the linear function in the double logarithmic plot was performed by using individually fitted *α* and *C* values in an alternative version of formula (3). In this case the term expressing spectral peak power *P*_*Peak*_*(f)* was omitted, and ln *P(f)* values were calculated for *f* values equalling 7.4, 10, 12.2, 13.5, 15 or 20 Hz. The goal of this step was the determination of the scale-free part of the spectrum at which the line cross of the y-axis is statistically independent from the slope of the line (*α*) across subjects.

### Estimation of the spectral peak frequencies

Spectral peak frequency was determined in the 9–18 Hz range, separately for each EEG recording location by automatically defining local maxima in mathematical terms. That is, we used the first derivative test in order to find the critical points, followed by the second derivative test to differentiate local maxima and minima. A spectral peak was accepted if the first order derivative was zero and the second order derivative was negative. Calculations were performed as follows: a second-degree polynomial curve fitting was performed using all sets of successive bin triplets (0.75 Hz), with an overlap of 2 bins (0.5 Hz) in the 9–18 Hz range resulting in equations of the following type:

4 $$P\left(f\right)= a{f}^{2}+bf+c$$

*P*: power; *f*: frequency (9–18 Hz); *a*, *b*, and *c*: fitted parameters.

The first derivative of these functions were calculated for each triplet, resulting in:

5 $${P}^{^{\prime}}\left(f\right)=2af+b$$

The slope of the function described in formula (5) is 2*a*, which was considered as the derivative at the middle of the triplets, resulting in the first derivative function of the spectra. The procedure was repeated for calculating the second derivatives: in this case the first order derivative function served as an input for fitting the quadratic polynomials.

Zero-crossings of the first derivatives were determined by spline interpolation (interpolating the series between the bins of 0.25 Hz). In addition, the second derivative was interpolated by the spline method at each detected zero crossing of the first derivatives. The cases which were characterized by the co-ocurrences of the two criteria below were considered as spectral peak frequencies:

6.1 $${P}^{{\prime}}\left(f\right)=0$$

6.2 $${P}{^{\prime\prime}}\left(f\right)<0$$

### Estimation of the spectral peak amplitudes

Spectral power at peak frequencies were estimated by spline interpolation of the double logarithmic plots of the power spectra. The spectral peak amplitude was then whitened by subtracting the estimated power based on the fitted linear function from the peak power containing both arrhythmic and rhythmic activity (Fig. 2):

7 $$\mathrm{ln}{P}_{Peak}\left(f\right)= \mathrm{ln}P\left(f\right)- \left(\mathrm{ln}C+ \alpha \mathrm{ln}f\right)$$

In order to avoid negative amplitudes due to the logarithmic scale, the power values were shifted for being all positive before this subtraction by adding a constant. This latter step was applied for the calculation of the amplitude measures only. As multiple spectral peaks were detected for some of the participants/EEG recording locations, the one with the largest amplitude was determined and used in this study. If no spectral peak was found in the spindle frequency range, peak values were considered as missing data (see Suppl. table 8). Data analysis was performed by MATLAB 9.5 (Mathworks Inc., https://www.mathworks.com).

### Antero-posterior changes in prevailing spectral peak frequency

Frequency measures of spectral peaks with maximal amplitudes (*P*_*Peak*_*(*_*fmaxPeak*_*)*) were analyzed in terms of antero-posterior changes as follows. First, we formed (para)sagittal regions by averaging *f*_*maxPeak*_ values in frontopolar (Fp1, Fp2), frontal (F3, F4, Fz), central (C3, C4, Cz), parietal (P3, P4, Pz), as well as occipital (O1, O2) recording locations. In the following, the regional means of *f*_*maxPeak*_ values were serially subtracted in consecutive antero-posterior regions as follows: frontal-frontopolar, central-frontal, parietal-central, occipital-parietal. Outputs express the antero-posterior shifts in *f*_*maxPeak*_ (Hz), with positive values indicating antero-posterior increases in frequency (Fig. 2). The successive frequency shifts were summed for each subject, whereas the results of this addition were averaged over the whole sample. In a separate analysis maximal frequency shifts were determined and localized in each subject, resulting a sample mean of maximal antero-posterior frequency shift and a topographical distribution of this shifts.

### FOOOF analyses

In order to test the convergent validity of our analyes we run separate analyses with a recently published method^23^. The same frequency range (2–48 Hz) was analyzed and the so-called knee-parameter, unique to the FOOOF-method was omitted, because the latter was specifically designed to describe the lowest frequency end of the spectrum (which we did not include in our analyses).

#### Statistical analyses

Goodness of fit of the linear to the equidistant log–log spectral data was assessed by Pearson product moment correlations, which were Fisher Z-transformed, averaged and back-transformed according to Silver and Dunlap^65^. Last, but not least the resulting average R-value were squared in order to determine the shared variance. Standard deviation (SD) was assessed from the Fisher-Z-transformed dataset, and the resulting value was back-transformed as well.

We used parametric tests (Pearson correlation, independent sample t-test) on normally distributed data and non-parametric tests (Spearman’s rank correlation, Mann–Whitney U test) when the distribution of the data was not Gaussian. The normality of the distributions was analysed by Shapiro–Wilk tests. In order to control Type 1 statistical errors due to multiple electrodes/hypothesis, we used a version of the Descriptive Data Analysis (DDA) protocol^37^ adapted for neurophysiological data^38,66^. This procedure tests the global null hypothesis (“all individual null hypotheses in the respective region are true”) at level 0.05, against the alternative that at least one of the null hypotheses is wrong. DDA considers the intercorrelations between the different electrodes and is based on defining Rüger’s areas^67^, which are sets of spatially contingent conventionally (descriptively) significant (*p* < 0.05) results. The global significance of the Rüger area means that at least 1/3 of the descriptive significances are significant at a *p* = 0.05/3 = 0.017 and/or 1/2 of the descriptive significances are significant at *p* = 0.05/2 = 0.025. We used both criteria simultaneously (the “and” operator) in this study. In order to obtain a better localization of regions with significant correlations, associations between NREM sleep EEG spindle frequency whitened spectral peak amplitudes and IQ were represented by significant probability maps^68^.

### Ethical statements

We confirm that we have read the Journal’s position on issues involved in ethical publication and affirm that this report is consistent with those guidelines.

## Supplementary Information

Supplementary Information

## Data Availability

All corrected power spectral data, as well as the fitted parameters and the program code used are available at https://osf.io/c487g/.
