# Supplementary material for: A set of composite, non-redundant EEG measures of NREM sleep based on the power law scaling of the Fourier spectrum
Source: Sci Rep. 2021 Jan 21;11:2041. doi: 10.1038/s41598-021-81230-7 (PMC7820008; doi:10.1038/s41598-021-81230-7)
Supplement: Supplementary file 1 — Supplementary Information [file 41598_2021_81230_MOESM1_ESM.docx]

**A set of composite, non-redundant EEG measures of NREM sleep based on the power law scaling of the Fourier spectrum**

Róbert Bódizs^1,2*^, Orsolya Szalárdy^1,3^, Csenge Horváth^1^, Péter P. Ujma^1,2^, Ferenc Gombos^4,5^, Péter Simor^1,6,7^, Adrián Pótári^5,8^, Marcel Zeising^9,10^, Axel Steiger^9^, Martin Dresler^11^

^1^Institute of Behavioural Sciences, Semmelweis University, Budapest, Hungary; ^2^Epilepsy Center, National Institute of Clinical Neurosciences, Budapest, Hungary; ^3^Institute of Cognitive Neuroscience and Psychology, Research Centre for Natural Sciences, Budapest, Hungary; ^4^Department of General Psychology, Pázmány Péter Catholic University, Budapest, Hungary; ^5^MTA‐PPKE Adolescent Development Research Group, Budapest, Hungary; ^6^Institute of Psychology, ELTE, Eötvös Loránd University, Budapest, Hungary; ^7^UR2NF, Neuropsychology and Functional Neuroimaging Research Unit at CRCN - Center for Research in Cognition and Neurosciences and UNI - ULB Neurosciences Institute, Université Libre de Bruxelles (ULB), Brussels, Belgium; ^8^Doctoral School of Psychology (Cognitive Science), Budapest University of Technology and Economics, Budapest, Hungary; ^9^Max Planck Institute of Psychiatry, Research Group Sleep Endocrinology, Munich, Germany; ^10^Centre of Mental Health, Klinikum Ingolstadt, Ingolstadt, Germany; ^11^Donders Institute for Brain, Cognition and Behaviour, Radboud University Medical Center, Nijmegen, The Netherlands

*** Correspondending author**

E-mail: [bodizs.robert@med.semmelweis-univ.hu](mailto:bodizs.robert@med.semmelweis-univ.hu)

Supplementary table 1. Descriptive statistics of spectral slopes and intercepts

| Rec. location | Valid N | Md_α_  (Q1–Q3) | Md_lnC_  (Q1–Q3) |
| --- | --- | --- | --- |
| Fp1 | 163 | -2.59  (-2.73 – -2.44) | 4.95  (4.47–5.40) |
| Fp2 | 171 | -2.62  (-2.74 – -2.45) | 5.02  (4.54–5.39) |
| F3 | 174 | -2.66  (-2.79 – -2.53) | 5.38  (4.86–5.75) |
| F4 | 173 | -2.66  (-2.81 – -2.53) | 5.41  (4.91–5.81) |
| Fz | 156 | -2.73  (-2.89 – -2.62) | 5.81  (5.21–6.20) |
| F7 | 153 | -2.47  (-2.62 – -2.35) | 4.40  (3.98–4.89) |
| F8 | 154 | -2.48  (-2.63 – -2.37) | 4.48  (4.07–4.91) |
| C3 | 174 | -2.62  (-2.77 – -2.50) | 5.15  (4.68–5.53) |
| C4 | 175 | -2.66  (-2.78 – -2.51) | 5.23  (4.74–5.61) |
| Cz | 156 | -2.70  (-2.82 – -2.56) | 5.78  (5.30–6.15) |
| P3 | 175 | -2.53  (-2.66 – -2.43) | 4.77  (4.37–5.16) |
| P4 | 175 | -2.53  (-2.68 – -2.42) | 4.77  (4.33–5.17) |
| T3 | 154 | -2.39  (-2.53 – -2.25) | 3.80  (3.34–4.28) |
| T4 | 156 | -2.40  (-2.52 – -2.26) | 3.90  (3.51–4.32) |
| T5 | 154 | -2.36  (-2.46 – -2.21) | 3.79  (3.29–4.20) |
| T6 | 155 | -2.35  (-2.46 – -2.21) | 3.75  (3.21–4.21) |
| O1 | 175 | -2.42  (-2.52 – -2.26) | 4.14  (3.80–4.74) |
| O2 | 174 | -2.42  (-2.51 – -2.29) | 4.20  (3.79–4.74) |

Md – median, Q1–Q3 – interquartile range


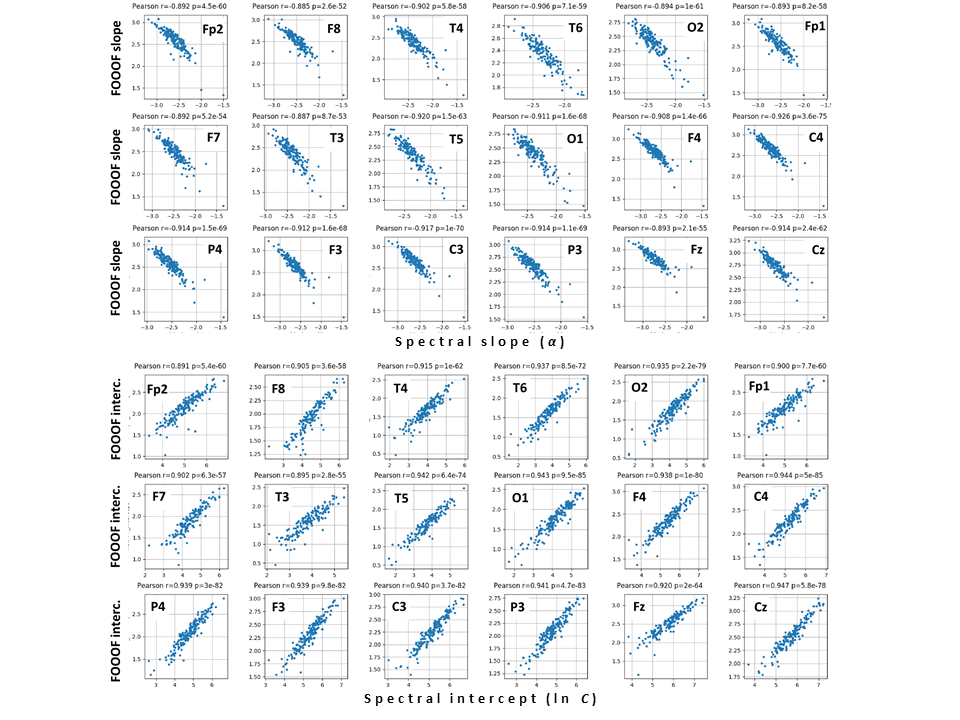


Supplementary figure 1. Testing the spectral slopes (*α*) and intercepts (ln *C*) against the respective outputs of the recently published fitting oscillations & one over f (FOOOF) method. Scatterplots indicate a reliable correspondence between the spectral slopes used in the present study (*α*) and the FOOOF slopes (lines 1–3), as well as between the spectral intercepts of the present study (ln *C*) and the FOOF intercepts (lines 4–6). Note that the negative correlations in lines 1–3 result from the different, but numerically equivalent mathematical description of the spectral slopes in the two methods: f*^α^* on the horizontal scale (where *α* is negative) and 1/f*^α^* (where *α* is positive).

Supplementary table 2. Descriptive statistics of spectral peak parameters

| Rec. location | Maximal peak | | | Secondary peak | | |
| --- | --- | --- | --- | --- | --- | --- |
|  | % valid obs. | Md f_maxPeak_  (Q1–Q3) | Md P_Peak_(f_maxPeak_)  (Q1–Q3) | % valid obs. | Md f_2ndPeak_  (Q1–Q3) | Md P_Peak_(f_2ndPeak_)  (Q1–Q3) |
| \| Fp1 \| \| --- \| | 92.07 | 11.55  (11.02–12.26) | 1.32  (0.94–1.70) | 10.98 | 9.91  (9.55–10.67) | 0.67  (0.34–1.17) |
| \| Fp2 \| \| --- \| | 90.69 | 11.52  (10.98–12.15) | 1.32  (0.98–1.69) | 9.30 | 9.85  (9.38–10.40) | 0.77  (0.50–1.10) |
| \| F3 \| \| --- \| | 95.42 | 12.33  (11.39–13.02) | 1.68  (1.37–2.03) | 24.00 | 10.19  (9.74–10.70) | 1.18  (0.72–1.49) |
| \| F4 \| \| --- \| | 95.40 | 12.47  (11.35–13.15) | 1.73  (1.40–2.01) | 28.74 | 10.22  (9.85–10.74) | 1.17  (0.75–1.42) |
| \| Fz \| \| --- \| | 96.79 | 12.73  (11.20–13.24) | 1.71  (1.43–2.08) | 39.74 | 10.30  (9.96–10.87) | 1.20  (0.81–1.43) |
| \| F7 \| \| --- \| | 89.54 | 11.85  (11.34–12.43) | 1.39  (0.97–1.74) | 14.38 | 9.88  (9.16–11.29) | 0.57  (0.30–0.94) |
| \| F8 \| \| --- \| | 91.55 | 11.82  (11.33–12.40) | 1.32  (0.98–1.68) | 11.69 | 9.86  (9.31–10.73) | 0.77  (0.60–1.02) |
| \| C3 \| \| --- \| | 98.85 | 13.39  (13.01–13.75) | 2.00  (1.63–2.28) | 40.00 | 10.56  (10.07–11.16) | 1.18  (0.89–1.37) |
| \| C4 \| \| --- \| | 98.29 | 13.41  (13.04–13.78) | 1.95  (1.68–2.22) | 36.93 | 10.63  (10.06–11.19) | 1.22  (0.99–1.49) |
| \| Cz \| \| --- \| | 99.35 | 13.50  (13.12–13.85) | 2.12  (1.80–2.42) | 50.64 | 10.50  (10.09–11.13) | 0.94  (0.72–1.38) |
| \| P3 \| \| --- \| | 100 | 13.49  (13.14–13.91) | 2.28  (1.87–2.63) | 23.30 | 10.21  (9.73–11.10) | 1.18  (0.78–1.40) |
| \| P4 \| \| --- \| | 99.43 | 13.52  (13.12–13.91) | 2.20  (1.80–2.54) | 25.57 | 10.47  (9.77–11.07) | 1.16  (0.88–1.34) |
| \| T3 \| \| --- \| | 81.16 | 12.85  (12.11–13.37) | 1.15  (0.88–1.50) | 17.53 | 10.35  (9.84–11.27) | 0.79  (0.52–1.16) |
| \| T4 \| \| --- \| | 83.97 | 12.94  (11.88–13.35) | 1.09  (0.83–1.42) | 20.51 | 10.92  (10.00–11.54) | 0.73  (0.49–1.09) |
| \| T5 \| \| --- \| | 98.70 | 13.40  (13.10–13.77) | 1.61  (1.29–1.89) | 16.88 | 10.70  (9.87–11.40) | 1.15  (0.87–1.36) |
| \| T6 \| \| --- \| | 97.41 | 13.44  (13.09–13.77) | 1.49  (1.21–1.77) | 19.35 | 10.99  (10.20–11.53) | 0.97  (0.73–1.26) |
| \| O1 \| \| --- \| | 98.86 | 13.45  (13.09–13.83) | 1.73  (1.35–2.16) | 9.09 | 9.55  (9.22–10.63) | 1.07  (0.63–1.29) |
| \| O2 \| \| --- \| | 98.85 | 13.45  (13.09–13.82) | 1.66  (1.29–2.09) | 6.29 | 9.84  (9.54–11.00) | 0.95  (0.55–1.28) |

Note that the largest interquartile range covering usual frequencies of both slow and fast sleep spindles in maximal spectral peak frequency was observed in the prefrontal region (recording locations F3, F4 and Fz). This implies that peak parameters indicate mixed slow and fast sleep spindle measures in this region. In addition, interquartile ranges anterior or posterior to this region are small, thus reflecting slow or fast sleep spindle activity, respectively.

Md – median, Q1–Q3 – interquartile range


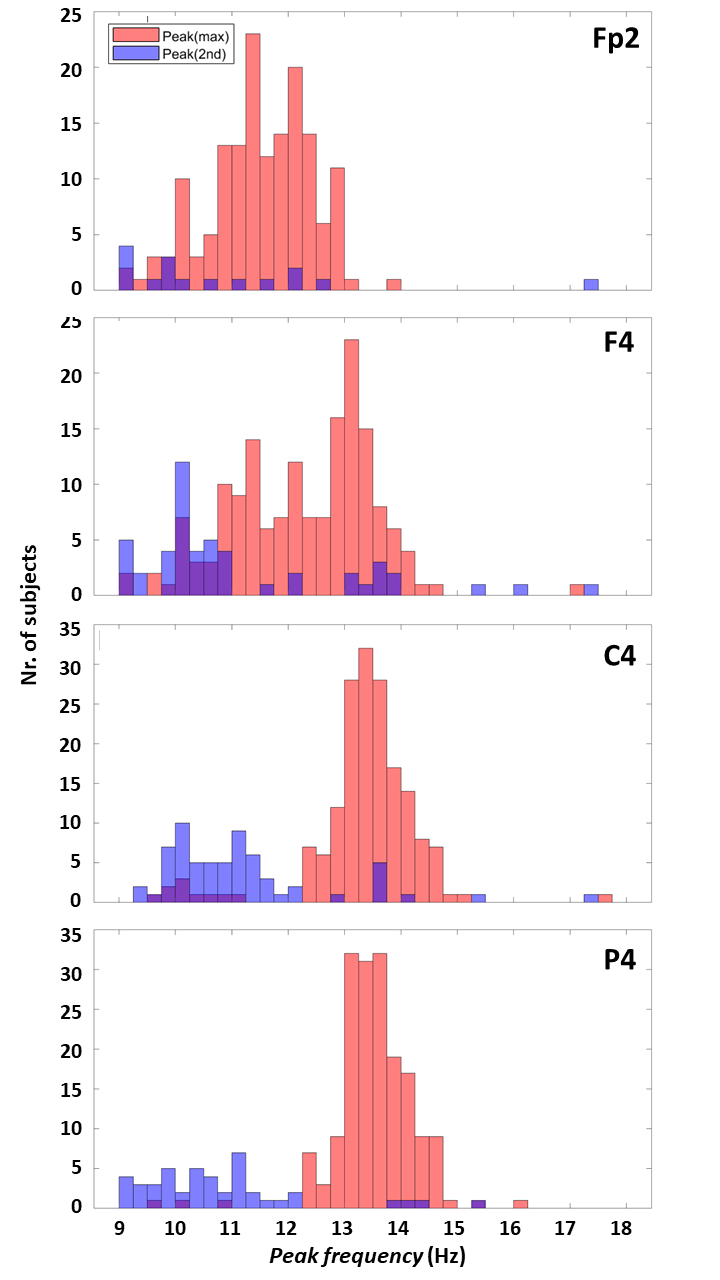


Supplementary figure 2. Histogram of maximal and secondary spectral peak frequencies. Note the lower median frequencies in frontal as compared to the central and parietal recording locations in terms of maximal spectral peaks (Md_maxPeak_Fp2_ = 11.52 Md_maxPeak_F4_ = 12.27, Md_maxPeak_C4_ = 13.33, Md_maxPeak_P4_ = 13.51), as well as the low occurrence rate of secondary peaks. Based on their nominal frequencies secondary peaks is rather alpha (~10 Hz) activity (medians are 9.85, 10.22, 10.63, 10.47 at recording locations Fp2, F4, C4, and P4, respectively).

Supplementary table 3. Correlations of age with spectral slopes, peak amplitudes and frequencies of NREM sleep EEG

| Recording location | a, slope (*α*) | | | b, peak amplitude (*P_Peak_(f_maxPeak)_*) | | | c, peak frequency (*f_maxPeak_*) | | |
| --- | --- | --- | --- | --- | --- | --- | --- | --- | --- |
|  | ρ | p | N | ρ | p | N | ρ | p | N |
| Fp1 | .38 | ***<.001*** | 163 | -.107 | .193 | 150 | -.22 | ***.006*** | 150 |
| Fp2 | .39 | ***<.001*** | 171 | -.035 | .669 | 155 | -.21 | ***.007*** | 155 |
| F3 | .39 | ***<.001*** | 174 | -.184 | ***.018*** | 166 | -.27 | ***<.001*** | 166 |
| F4 | .42 | ***<.001*** | 173 | -.17 | *.029* | 165 | -.25 | ***.001*** | 165 |
| Fz | .44 | ***<.001*** | 156 | -.288 | ***<.001*** | 151 | -.37 | ***<.001*** | 151 |
| F7 | .36 | ***<.001*** | 153 | -.105 | .223 | 137 | -.29 | ***.001*** | 137 |
| F8 | .38 | ***<.001*** | 154 | -.002 | .978 | 141 | -.21 | ***.010*** | 141 |
| C3 | .38 | ***<.001*** | 174 | -.328 | ***<.001*** | 172 | -.05 | .46 | 172 |
| C4 | .39 | ***<.001*** | 175 | -.32 | ***<.001*** | 172 | -.04 | .586 | 172 |
| Cz | .35 | ***<.001*** | 156 | -.409 | ***<.001*** | 155 | -.06 | .44 | 155 |
| P3 | .34 | ***<.001*** | 175 | -.23 | ***.002*** | 175 | .10 | .16 | 175 |
| P4 | .37 | ***<.001*** | 175 | -.227 | ***.003*** | 174 | .08 | .253 | 174 |
| T3 | .39 | ***<.001*** | 154 | -.141 | .116 | 125 | -.14 | .119 | 125 |
| T4 | .39 | ***<.001*** | 156 | -.125 | .154 | 131 | -.30 | ***.001***­­­­­­ | 131 |
| T5 | .33 | ***<.001*** | 154 | -.176 | *.030* | 152 | .01 | .861 | 152 |
| T6 | .37 | ***<.001*** | 155 | -.213 | ***.009*** | 151 | -.00 | .988 | 151 |
| O1 | .29 | ***<.001*** | 175 | -.127 | .097 | 173 | .10 | .179 | 173 |
| O2 | .33 | ***<.001*** | 174 | -.132 | .085 | 172 | .11 | .152 | 172 |

a, Spearman rank correlations (ρ) between age and spectral exponents (α) of NREM sleep EEG. Note the significance of all correlations at the descriptive level of significance (p < .05), as well as at both of the new critical p levels corresponding to p < .025 and p < .017. The minimum criteria of a significant Rüger’s area is 10 out of 18 descriptive significances to meet the p < .025 and 7 out of 18 descriptive significances to meet the p < .017 criteria.

b, Correlations between age and whitened maximal spectral peak amplitude P_Peak_(f_maxPeak_) of NREM sleep EEG spindle frequency activity (9 Hz < f < 18 Hz). Note the descriptive significance of 9 correlations forming a large Rüger area in the bilateral fronto-centro-parietal and posterior temporal region. The minimum criteria for a significant Rüger area is at least 5 correlations to be significant at p < .025 and 4 at p < .017. Here we found 5 and 4 correlations meeting this criteria, respectively. Thus, the Rüger area can be considered as a global zone of significance.

c, Correlations between age and NREM sleep EEG maximal spectral peak frequencies in the spindle range (f_maxPeak_). Note the descriptive significance of 8 negative correlations forming a Rüger area in the bilateral frontal and right temporal region. The minimum criteria for a significant Rüger area is at least 5 correlations to be significant at p < .025 and 3 at p < .017. Here we found 8 for both, thus, the Rüger area can be considered as a global zone of significance. *p < .05*; ***p < .025***; ***p < .017***

Supplementary table 4. Women vs men differences in NREM sleep EEG spectral intercepts and whitened peak amplitudes in the spindle range

| Recording location | a, intercept (ln *C*) | | | | | | b, peak amplitude (*P_Peak_(f_maxPeak_)*) | | | | | |
| --- | --- | --- | --- | --- | --- | --- | --- | --- | --- | --- | --- | --- |
|  | U  (η^2^) | p | N_♀_ | Md_♀_  (Q1–Q3)_♀_ | N_♂_ | Md_♂_  (Q1–Q3)_♂_ | t  (d) | p | N_♀_ | _♀_  (CI 95%)_♀_ | N_♂_ | _♂_  (CI 95%)_♂_ |
| Fp1 | 2455  (.050) | ***.004*** | 77 | 5.11  (4.63–5.55) | 86 | 4.84  (4.32–5.23) | -.09  (-.015) | .924 | 67 | 1.37  (1.21–1.52) | 83 | 1.37  (1.26–1.48) |
| Fp2 | 2682  (.052) | ***.003*** | 81 | 5.14  (4.73–5.57) | 90 | 4.87  (4.43–5.32) | .51  (.083) | .619 | 68 | 1.40  (1.25–1.54) | 87 | 1.36  (1.26–1.45) |
| F3 | 2658  (.064) | ***.001*** | 81 | 5.53  (5.06–5.94) | 93 | 5.22  (4.79–5.60) | .42  (.066) | .675 | 75 | 1.73  (1.60–1.85) | 91 | 1.69  (1.60–1.79) |
| F4 | 2503  (.080) | ***<.001*** | 81 | 5.58  (5.25–6.03) | 92 | 5.28  (4.83–5.68) | 1.00  (.156) | .317 | 76 | 1.76  (1.64–1.87) | 89 | 1.68  (1.58–1.78) |
| Fz | 1997  (.082) | ***.001*** | 69 | 6.04  (5.41–6.44) | 87 | 5.61  (5.08–6.00) | .83  (.136) | .405 | 66 | 1.80  (1.67–1.93) | 85 | 1.73  (1.63–1.83) |
| F7 | 2295  (.030) | *.031* | 67 | 4.58  (4.15–4.95) | 86 | 4.28  (3.93–4.73) | 1.47  (.254) | .162 | 59 | 1.47  (1.30–1.63) | 78 | 1.33  (1.23–1.43) |
| F8 | 2382  (.026) | *.046* | 69 | 4.56  (4.16–5.01) | 85 | 4.44  (4.05–4.74) | .71  (.120) | .493 | 63 | 1.38  (1.23–1.53) | 78 | 1.32  (1.22–1.42) |
| C3 | 2345  (.106) | ***<.001*** | 81 | 5.36  (4.93–5.81) | 93 | 4.98  (4.55–5.26) | -.05  (-.008) | .953 | 80 | 1.98  (1.88–2.09) | 92 | 1.99  (1.88–2.10) |
| C4 | 2483  (.090) | ***<.001*** | 81 | 5.41  (5.03–5.86) | 94 | 5.04  (4.62–5.40) | .22  (.034) | .822 | 79 | 1.98  (1.87–2.08) | 93 | 1.96  (1.85–2.07) |
| Cz | 1869  (.105) | ***<.001*** | 69 | 5.98  (5.49–6.40) | 87 | 5.58  (5.20–5.96) | .43  (.070) | .665 | 68 | 2.14  (2.02–2.26) | 87 | 2.10  (1.98–2.22) |
| P3 | 2467  (.092) | ***<.001*** | 81 | 4.97  (4.53–5.47) | 94 | 4.69  (4.26–4.93) | -.39  (-.059) | .692 | 81 | 2.24  (2.12–2.35) | 94 | 2.27  (2.15–2.39) |
| P4 | 2423  (.098) | ***<.001*** | 81 | 5.04  (4.46–5.43) | 94 | 4.56  (4.20–4.99) | -.70  (-.106) | .483 | 81 | 2.14  (2.02–2.25) | 93 | 2.20  (1.85–2.07) |
| T3 | 2270  (.036) | ***.019*** | 67 | 3.99  (3.46–4.46) | 87 | 3.72  (3.31–4.18) | 1.02  (.184) | .306 | 55 | 1.25  (1.12–1.38) | 70 | 1.16  (1.06–1.27) |
| T4 | 2428  (.027) | *.041* | 69 | 4.02  (3.61–4.43) | 87 | 3.83  (3.46–4.20) | .98  (.173) | .327 | 57 | 1.19  (1.06–1.32) | 74 | 1.11  (1.02–1.21) |
| T5 | 2172  (.049) | ***.006*** | 68 | 3.92  (3.45–4.43) | 86 | 3.67  (3.11–4.01) | -1.09  (-.178) | .275 | 68 | 1.54  (1.43–1.65) | 84 | 1.63  (1.52–1.73) |
| T6 | 2021  (.074) | ***.001*** | 68 | 3.91  (3.54–4.41) | 87 | 3.65  (3.06–4.01) | -.46  (-.075) | .646 | 66 | 1.47  (1.36–1.58) | 85 | 1.50  (1.40–1.61) |
| O1 | 2182  (.135) | ***<.001*** | 81 | 4.51  (4.00–4.94) | 94 | 3.97  (3.44–4.37) | -1.07  (-.163) | .285 | 80 | 1.69  (1.57–1.80) | 93 | 1.78  (1.66–1.90) |
| O2 | 2102  (.145) | ***<.001*** | 81 | 4.53  (4.12–4.96) | 93 | 4.04  (3.54–4.36) | -1.93  (-.295) | .054 | 80 | 1.59  (1.47–1.70) | 92 | 1.74  (1.63–1.86) |

a. Mann-Whitney U tests indicate higher spectral intercepts (ln *C* values) in the female (♀) as compared to the male (♂) subgroup. Descriptive significance was observed at all (18) recording locations. Minimum criteria of a significant Rüger area requires at least 10 of these p values to be lower than .025 and 7 of them to be less than .017. Observed values are 15 and 14, respectively. Thus, the Rüger area characterizing the sex differences in NREM sleep EEG spectral intercepts is significant. Md – median; *p < .05*; ***p < .025***; ***p < .017***.

b. Independent sample t-tests indicate no significant sex differences in whitened maximal spectral peak amplitudes of the sleep spindle range of NREM sleep EEG (*P_Peak_(f_maxPeak_)*). d – Cohen d; CI 95%: Confidence interval with 95% limits.

Supplementary table 5. Correlation of whitened spectral peak amplitudes with IQ

| Recording location | Entire sample | | | Women | | | Men | | |
| --- | --- | --- | --- | --- | --- | --- | --- | --- | --- |
|  | r | p | N | r_♀_ | p_♀_ | N_♀_ | r_♂_ | p_♂_ | N_♂_ |
| Fp1 | 0.11 | 0.18 | 127 | .24 | .067 | 55 | .02 | .830 | 72 |
| Fp2 | 0.06 | 0.49 | 132 | .16 | .231 | 55 | .01 | .985 | 77 |
| F3 | 0.04 | 0.56 | 140 | .15 | .216 | 62 | -.01 | .920 | 78 |
| F4 | 0.05 | 0.52 | 139 | .17 | .178 | 63 | -.01 | .989 | 76 |
| Fz | 0.12 | 0.18 | 125 | .22 | .101 | 53 | .08 | .499 | 72 |
| F7 | 0.04 | 0.65 | 115 | .11 | .432 | 48 | .02 | .858 | 67 |
| F8 | 0.01 | 0.88 | 118 | .02 | .880 | 52 | .03 | .759 | 66 |
| C3 | 0.14 | 0.08 | 146 | .32 | ***.006*** | 67 | .02 | .842 | 79 |
| C4 | 0.15 | 0.06 | 147 | .34 | ***.004*** | 66 | .03 | .746 | 81 |
| Cz | 0.17 | *0.04* | 129 | .34 | ***.010*** | 55 | .09 | .426 | 74 |
| P3 | 0.07 | 0.39 | 149 | .26 | *.030* | 68 | -.07 | .531 | 81 |
| P4 | 0.08 | 0.28 | 149 | .28 | ***.020*** | 68 | -.05 | .627 | 81 |
| T3 | 0.18 | 0.06 | 103 | .32 | *.030* | 45 | .09 | .484 | 58 |
| T4 | 0.06 | 0.47 | 108 | .23 | .118 | 46 | -.04 | .718 | 62 |
| T5 | 0.09 | 0.29 | 127 | .19 | .147 | 55 | .03 | .771 | 72 |
| T6 | 0.08 | 0.33 | 127 | .15 | .266 | 54 | .06 | .609 | 73 |
| O1 | 0.09 | 0.22 | 148 | .22 | .061 | 67 | .03 | .731 | 81 |
| O2 | 0.10 | 0.21 | 147 | .23 | .057 | 67 | .01 | .888 | 80 |

The Rüger area at the centroparietal-left temporal region characterized by descriptive significances (p < .05) in the female subgroup (♀) remained significant after the control for multiple testing (4/6 correlations are significant at .05/2 and 3/6 at .05/3). No significant correlations of whitened spectral peak amplitude and IQ were found in the male subgroup (♂).*p < .05*; ***p < .025***; ***p < .017***.

Supplementary table 6. Data on the lack of sex differences in spectral slopes and the relationship between spectral slopes and intercepts

| Recording location | a, sex differences in *α* | | | | | | b, α vs ln *C_0_* | | |
| --- | --- | --- | --- | --- | --- | --- | --- | --- | --- |
|  | t  (d) | p | N_♀_ | (CI 95%) | N_♂_ | (CI 95%) | r | p | N |
| Fp1 | 1.452  (.228) | .148 | 77 | -2.613  (-2.66–-2.56) | 86 | -2.563  (-2.61–-2.51) | -.83 | ***<.001*** | 163 |
| Fp2 | 1.477  (.226) | .141 | 81 | -2.624  (-2.67–-2.57) | 90 | -2.575  (-2.62–-2.52) | -.84 | ***<.001*** | 171 |
| F3 | 1.578  (.240) | .116 | 81 | -2.682  (-2.72–-2.63) | 93 | -2.629  (-2.67–-2.57) | -.83 | ***<.001*** | 174 |
| F4 | 1.585  (.241) | .114 | 81 | -2.689  (-2.73–-2.64) | 92 | -2.634  (-2.68–-2.58) | -.85 | ***<.001*** | 173 |
| Fz | 1.308  (.211) | .192 | 69 | -2.760  (-2.80–-2.71) | 87 | -2.713  (-2.76–-2.66) | -.85 | ***<.001*** | 156 |
| F7 | 1.307  (.213) | .192 | 67 | -2.510  (-2.56–-2.45) | 86 | -2.460  (-2.51–-2.40) | -.85 | ***<.001*** | 153 |
| F8 | 1.276  (.207) | .203 | 69 | -2.512  (-2.56–-2.46) | 85 | -2.465  (-2.51–-2.41) | -.83 | ***<.001*** | 154 |
| C3 | 2.044  (.311) | *.042* | 81 | -2.658  (-2.69–-2.61) | 93 | -2.594  (-2.63–-2.54) | -.78 | ***<.001*** | 174 |
| C4 | 1.558  (.236) | .120 | 81 | -2.664  (-2.70–2.62) | 94 | -2.612  (-2.66–-2.56) | -.81 | ***<.001*** | 175 |
| Cz | .878  (.142) | .381 | 69 | -2.701  (-2.74–-2.65) | 87 | -2.672  (-2.71–-2.62) | -.81 | ***<.001*** | 156 |
| P3 | 1.407  (.213) | .161 | 81 | -2.558  (-2.59–-2.51) | 94 | -2.516  (-2.55–-2.47) | -.79 | ***<.001*** | 175 |
| P4 | 1.398  (.212) | .163 | 81 | -2.553  (-2.59–-2.51) | 94 | -2.510  (-2.55–-2.46) | -.80 | ***<.001*** | 175 |
| T3 | 1.112  (.181) | .267 | 67 | -2.404  (-2.45–-2.35) | 87 | -2.362  (-2.41–-2.30) | -.80 | ***<.001*** | 154 |
| T4 | 1.031  (.166) | .303 | 69 | -2.400  (-2.45–-2.34) | 87 | -2.361  (-2.41–-2.31) | -.81 | ***<.001*** | 156 |
| T5 | .951  (.154) | .343 | 68 | -2.352  (-2.40–-2.30) | 86 | -2.317  (-2.36–-2.26) | -.80 | ***<.001*** | 154 |
| T6 | 1.358  (.220) | .176 | 68 | -2.359  (-2.40–-2.30) | 87 | -2.312  (-2.35–-2.26) | -.76 | ***<.001*** | 155 |
| O1 | 1.991  (.302) | *.048* | 81 | -2.425  (-2.46–-2.38) | 94 | -2.362  (-2.40–-2.31) | -.79 | ***<.001*** | 175 |
| O2 | 2.038  (.310) | *.043* | 81 | -2.430  (-2.47–-2.38) | 93 | -2.369  (-2.41–-2.32) | -.77 | ***<.001*** | 174 |

a. Sex differences in NREM sleep EEG spectral slopes as revealed by independent sample t-tests. The 3 descriptive significances do not survive the control of Type I error. b. The correlation of NREM sleep EEG spectral slopes (*α*) and intercepts at ln *f* = 0 (ln *C_0_*). Correlations are significant over the whole registered area and survive the control of multiple testing. Fisher z-transformed, averaged and back-transformed correlation value is -0.811. *p < .05*; ***p < .025***; ***p < .017***.

Supplementary table 7. Technical details of the recordings in different subsamples included in the present investigation

| Subsample | Recording apparatus | Precision (bit) | Hardware (firmware) filtering (Hz) | Sampling frequency (Hz/channel) | N |
| --- | --- | --- | --- | --- | --- |
| Budapest-I | Flat Style Lamont Headbox, HBX32-SLP preamplifier | 12 | 0.5–70 | 249 | 43 |
| Budapest-II | Brain-Quick BQ132S Headbox and EEG Amplifier | 12 | 0.33–1500  (0.33–450) | 4096  (decimated to 1024 Hz after filtering by firmware) | 19 |
| Münich | Comlab 32 Digital Sleep Lab | 8 | 0.53–70 Hz | 250 | 113 |

Supplementary table 8. The number of missing/artefactual records (EEG) and peak power values (P_Peak_), separately for each recording location

|  | Fp1 | Fp2 | F3 | F4 | Fz | F7 | F8 | C3 | C4 | Cz | P3 | P4 | T3 | T4 | T5 | T6 | O1 | O2 |
| --- | --- | --- | --- | --- | --- | --- | --- | --- | --- | --- | --- | --- | --- | --- | --- | --- | --- | --- |
| EEG | 19 | 13 | 10 | 10 | 28 | 31 | 30 | 10 | 9 | 28 | 9 | 9 | 30 | 28 | 30 | 29 | 9 | 10 |
| P_Peak_ | 32 | 29 | 18 | 18 | 33 | 45 | 43 | 12 | 12 | 29 | 9 | 10 | 59 | 53 | 32 | 33 | 11 | 12 |
